# Efficient Intestinal Digestion and On Site Tumor‐Bioactivation are the Two Important Determinants for Chylomicron‐Mediated Lymph‐Targeting Triglyceride‐Mimetic Docetaxel Oral Prodrugs

**DOI:** 10.1002/advs.201901810

**Published:** 2019-10-25

**Authors:** Chutong Tian, Jingjing Guo, Gang Wang, Bingjun Sun, Kexin Na, Xuanbo Zhang, Zhuangyan Xu, Maosheng Cheng, Zhonggui He, Jin Sun

**Affiliations:** ^1^ Department of Pharmaceutics Wuya College of Innovation Shenyang Pharmaceutical University No. 103, Wenhua Road Shenyang 110016 China; ^2^ School of Pharmacy Guang Xi University of Chinese Medicine Wuhe Rode Nanning 530200 China; ^3^ Key Laboratory of Structure‐Based Drug Design & Discovery of Ministry of Education Shenyang Pharmaceutical University Shenyang 110016 China; ^4^ Department of Pharmaceutics Wuya College of Innovation Shenyang Pharmaceutical University Shenyang 110016 P. R. China; ^5^ Municipal Key Laboratory of Biopharmaceutics Wuya College of Innovation Shenyang Pharmaceutical University Shenyang 110016 P. R. China

**Keywords:** docetaxel, lymph transport, oral chemotherapy, reduction‐sensitive, triglyceride‐mimetic prodrugs

## Abstract

The oral absorption of chemotherapeutical drugs is restricted by poor solubility and permeability, high first‐pass metabolism, and gastrointestinal toxicity. Intestinal lymphatic transport of lipophilic prodrugs is a promising strategy to improve the oral delivery efficiency of anticancer drugs via entrapment into a lipid formulation and to avoid first‐pass metabolism. However, several basic principles have still not been clarified, such as intestinal digestibility and stability and on‐site tumor bioactivation. Herein, triglyceride‐mimetic prodrugs of docetaxel (DTX) are designed by conjugating them to the sn‐2 position of triglyceride (TG) through different linkage bonds. The role of intestinal digestion in oral absorption of TG‐like prodrugs is then investigated by introducing significant steric‐hindrance α‐substituents into the prodrugs. It is surprisingly found that poor intestinal digestion leads to an unsatisfactory bioavailability but efficient intestinal digestion of TG‐like prodrugs with a less steric‐hindrance linkage (DTX‐S‐S‐TG) facilitating oral absorption. Moreover, it is found that the TG‐like reduction‐sensitive prodrug (DTX‐S‐S‐TG) has good stability during intestinal transport and blood circulation, and on‐demand release of docetaxel at the tumor site, leading to a significantly improved antitumor efficiency with negligible gastrointestinal toxicity. In summary, the chylomicron‐mediated lymph‐targeting triglyceride‐mimetic oral prodrug approach provides a good foundation for the development of oral chemotherapeutical formulations.

## Graphical abstract

In this work, bioinspired triglyceride‐mimetic docetaxel prodrugs are designed to improve oral absorption and antitumor efficiency of docetaxel. The important role of intestinal digestibility in triglyceride‐like prodrug is studied systematically. Moreover, a reduction‐sensitive disulfide bond is implemented to realize on‐demand tumor site release of chemotherapeutical drug, leading to good tumor inhibition and reduced gastrointestinal toxicity.

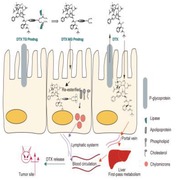

## 1 Introduction

Oral chemotherapy is a preferred administration route with distinct advantages: it is conducive to patients' compliance,^1^ easy to administer and it engenders a high quality of life.^2^ However, the current state of oral chemotherapy is far from satisfactory due to its poor bioavailability and serious gastrointestinal (GI) toxicity. A typical example is docetaxel (DTX), the first‐line drug used for breast cancer treatment in the clinic.^3^ The oral bioavailability of DTX is greatly limited by its poor water solubility and low permeability.^4^ Many strategies have been developed to facilitate the oral delivery of DTX such as micelles,^5^ microemulsions,^6^ solid lipid nanoparticles,^7^ and lecithin nanoparticles.^8^ Efflux by P‐gp pump and extensive first‐pass effect are usually the leading causes of its poor bioavailability.^9^ Therefore, P‐gp/CYP450 inhibitors such as ritonavir and cyclosporin A are coadministered with DTX to improve its oral delivery efficiency.^10^ However, no available oral docetaxel product has been on the market so far. All of the studies have their own limitations, including significant GI toxicity caused by drug leakage in the GI tract or the immunosuppression caused by P‐gp/CYP450 inhibitors.^11^

Triglycerides (TG) are difficult for the intestine to directly absorb by intestine. They are first digested into mono‐ and diglycerides and free fatty acids, but not free glycerol, by pancreatic lipase. The resulting mixtures of di‐ and monoglycerides and free fatty acids, together with bile salts and phospholipids, form mixed micelles, which facilitate the contents of the mixed micelles entering the enterocytes. Once absorbed into the enterocytes, they are re‐esterified into TG and encapsulated in chylomicrons that are transported into the lymphatic circulation.^12^ This means that the lymph‐targeting triglyceride‐mimetic prodrugs could bypass the liver for a decreased first‐pass effect.^13^ Recently, Porter and co‐workers designed glyceride‐mimetic prodrugs of testosterone with the introduction of a self‐immolative spacer, which improved the oral bioavailability of testosterone up to 90‐fold.^14^ However, the importance of intestinal digestibility was not studied systematically. Moreover, for oral chemotherapy, an intact prodrug form before entering the blood circulation is important for decreased GI toxicity and then tumor‐triggered bioactivation is essential to elicit a good anticancer efficacy.^15^ The difference between normal cells and tumor cells on reduction condition could be exploited to develop reductive‐responsive prodrugs.^16^

**Figure 1:**
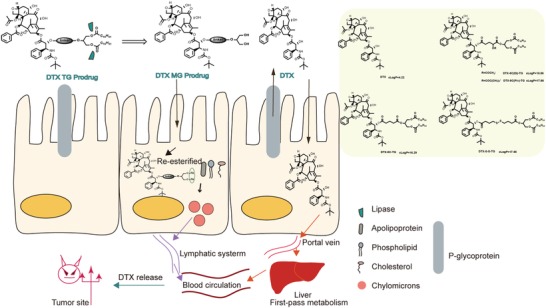
Oral absorption mechanisms of DTX or TG‐mimetic prodrugs after oral administration and chemical structures of DTX and its triglyceride‐mimetic prodrugs.

Herein, bioinspired triglyceride‐mimetic DTX prodrugs have been designed in which the DTX is covalently conjugated to the sn‐2 position of long‐chain triglyceride via different linkage bonds. As shown in **Figure** **1**, the triglyceride‐mimetic prodrugs show distinct advantages in the field of oral chemotherapy: (i) the fatty chain moiety of triglyceride‐mimetic prodrugs increases their oil‐solubility and help in high loading efficiency of prodrugs into an emulsion; and (ii) the first‐pass metabolism can be avoided by the lymph‐targeting prodrugs transport. Moreover, in order to investigate the role of intestinal digestion in the oral absorption of triglyceride‐mimetic prodrugs, significant steric‐hindrance α‐substituents were introduced into linkers bridging docetaxel with the triglyceride skeleton (DTX‐5C(Et)‐TG and DTX‐5C(Piv)‐TG, Figure 1) to hinder lipase accessibility. In addition, a reduction‐sensitive disulfide bond was inserted into the linker (DTX‐S‐S‐TG, Figure 1) to achieve specific drug release in tumor cells for decreased system toxicity and to maintain the good prodrug stability before entering the blood circulation in order to reduce GI toxicity.

## 2 Results and Discussion

### 2.1 Design and Synthesis of Prodrugs

DTX‐5C‐TG was synthesized by coupling DTX to triglyceride skeleton via a C5 linker. Significant steric‐hindrance α‐substituents were introduced into the C5 linker to investigate the influence of lipase accessibility on intestinal digestion and DTX‐5C(Et)‐TG and DTX‐5C(Piv)‐TG were synthesized. A novel reduction‐sensitive DTX triglyceride mimetic prodrug (DTX‐S‐S‐TG) was designed by inserting a disulfide bond into the carbon chain linkage. Reduction‐nonsensitive DTX‐5C‐TG was designed as a control. Their chemical structures (Figure 1; Figure S1, Supporting Information) were confirmed by mass spectra, ^1^H NMR, ^13^C NMR and infrared spectroscopy as shown in Figures S2–S14, Supporting Information.

### 2.2 Characterization of Chylomicron‐Like Emulsions

Four prodrug‐loaded chylomicron‐like emulsions containing bile salt and lecithin were prepared, aiming to improve lipase activation^17^ and to protect the prodrugs from breakdown in the acidic condition of the stomach. The particle size, polydispersity index, zeta potential, and encapsulation efficiency of the prepared emulsions were listed in **Table** **1**. As shown in Table 1 and Figure S21, Supporting Information, the prepared emulsions showed subsphaeroidal‐shaped structures and exhibited a uniform particle size distribution, with the average diameter around 210 nm according to Malvern laser particle size analyzer and TEM. A negative zeta potential ranging from −40 to −36 mv was shown as well. The EE (%) of the TG prodrug emulsions were all above 90% due to the high oil solubility of the prodrugs. In contrast, the rigid planar‐structure of DTX limited its encapsulation, leading to a much lower EE ratio (67.0%). These results suggested that conjugating DTX with a triglyceride structure framework increased its lipid affinity.

**Table 1:** Characterization of emulsions (data are represented as mean ± SD, *n* = 3)

Formulations	Size [nm]	PDI	Zeta potential [mv]	EE [%]
DTX	213 ± 1.82	0.173 ± 0.05	−41.3 ± 1.49	67.0 ± 3.45
DTX‐5C‐TG	204 ± 1.25	0.188 ± 0.02	−36.5 ± 3.84	93.0 ± 0.63
DTX‐5C(Et)‐TG	223 ± 6.69	0.166 ± 0.02	−36.7 ± 1.40	96.8 ± 2.92
DTX‐S‐S‐TG	212 ± 3.03	0.164 ± 0.04	−45.5 ± 3.84	97.6 ± 1.39
DTX‐5C(Piv)‐TG	212 ± 2.26	0.172 ± 0.01	−45.2 ± 4.24	98.8 ± 0.25

### 2.3 Stability Studies

#### 2.3.1 Stability in Simulated Gastric Fluid (SGF)

**Figure 2:**
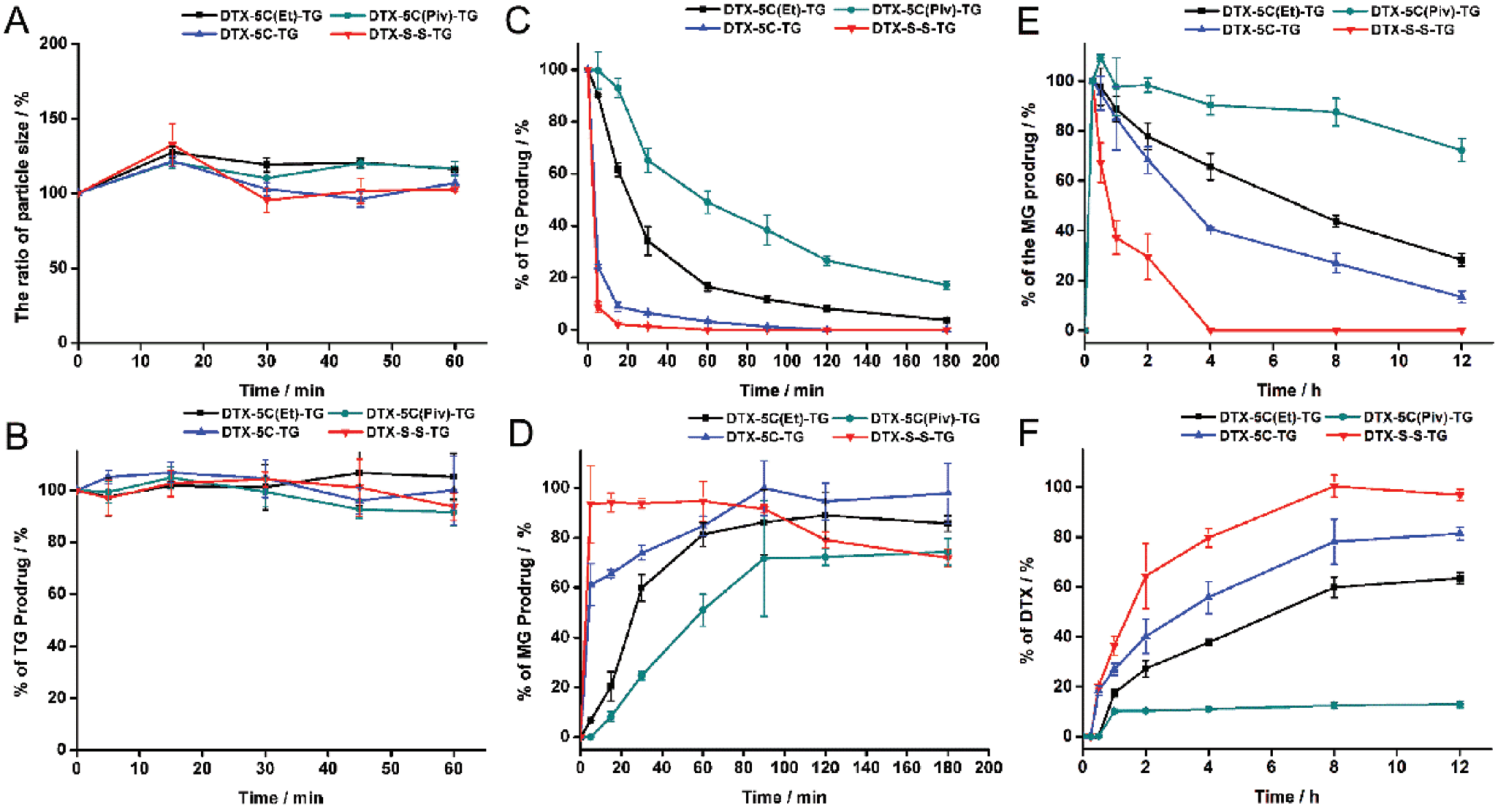
Stability of TG‐like prodrugs A) Physical stability of chylomicron‐like emulsions in SGF. B) Chemical stability of TG‐like prodrugs in SGF. C) Chemical stability of TG‐like prodrugs in bile‐pancreatic juice. D) Generation of MG prodrugs after incubation with bile‐pancreatic juice. E) Production of % MG prodrugs of all TG prodrugs in the plasma supplying with lipoprotein lipase (LPL). F) Production of DTX of all TG prodrug incubation in the plasma supplying with LPL (data are represented as mean ± SD, *n* = 3).

As shown in **Figure** **2**A, the four prodrug emulsions appeared to be physically stable in SGF over 1 h with a negligible increase in particle size. As well, all of the prodrugs showed a high chemical stability in SGF (Figure 2B). These results indicated that the four prodrug emulsions could maintain good physical and chemical stability, with no active parent drug leakage in the stomach, which may avoid gastric toxicity.

#### 2.3.2 Simulated Intestinal Digestion

Dietary TG is difficult for the intestine to absorb and must be hydrolyzed into monoglycerides and fatty acids by pancreatic lipase along with the effect of bile salts in the intestine for oral absorption. Evaluating the hydrolysis degree of triglyceride‐mimetic DTX prodrugs and the accumulation of monoglyceride derivatives of DTX are essential steps in determining the oral absorption potential of the TG‐like prodrugs. As shown in Figure 2C, the DTX‐S‐S‐TG prodrug was digested rapidly with only trace amounts left after 5 min of incubation in the bile‐pancreatic juice, followed by DTX‐5C‐TG, DTX‐5C(Et)‐TG, and DTX‐5C‐(Piv)‐TG. The DTX‐5C‐(Piv)‐TG prodrug showed the poorest intestinal digestion rate due to its bulky α‐substituents that sterically hinder pancreatic lipase accessibility. For the digestion products of the TG‐like prodrugs, MG derivatives of DTX (MG‐like prodrugs) in the simulated digesta were confirmed by mass spectra in Figure S22, Supporting Information and were named as DTX‐5C(Et)‐MG, DTX‐5C(Piv)‐MG, DTX‐5C‐MG, and DTX‐S‐S‐MG. The generated 2‐monoglyceride derivatives could form a series of colloidal structures that could cross the unstirred water layer (UWL) and be absorbed into enterocytes more easily. The amounts of the MG derivatives of TG‐like prodrugs were shown in Figure 2D. DTX‐S‐S‐MG derivative from DTX‐S‐S‐TG reached 100% in a few minutes but the DTX‐5C(Piv)‐MG derivative from DTX‐5C(Piv)‐TG was only 74% after 3 h of incubation in bile‐pancreatic juice. Clearly, the production rate of the MG derivatives was in a good agreement with the digestion rate of the TG‐like prodrugs.

Additionally, after intestinal digestion, the levels of TG‐like prodrugs and MG‐like prodrugs in mixed micellar phase (aqueous phase) were determined. After 3 h digestion, the digesta was centrifuged to be separated into three phases, i.e., the sedimental phase, the micellar phase, and the oil phase. Then the concentrations of MG or TG‐like prodrugs in raw digesta and micellar phase were measured, respectively. As shown in Figure S23A, Supporting Information, the micellar phase partition of the MG‐like prodrugs was surprisingly high (up to 100%) for all prodrugs. For the TG‐like prodrugs, DTX‐5C(Piv)‐TG and DTX‐5C(Et)‐TG showed about 40.2% and 69.8% partition into the micellar phase. By contrast, there was no detection of TG‐like prodrugs both in the raw digesta or the micellar phase after digestion for other two prodrugs, suggesting nearly complete intestinal digestion.

The particle size of the mix micelles was determined. As shown in Figure S23B, Supporting Information, the diameters of the micelles formed in the DTX‐S‐S‐TG prodrug emulsion and the DTX‐5C‐TG prodrug emulsion group, were smaller than those of the other two groups, which were not digested completely. The MG‐like prodrugs digested from the TG‐like prodrugs could be incorporated fully with other lipid digestion products to assemble into the small mixed micelles around 90 nm, which could diffuse across UML easily (Figure S23C, Supporting Information). However, the undigested TG‐like prodrugs precipitated in the intestine mostly and the residuals were assembled into larger micelles or vesicles. These large particles may be not easy to pass through UML (Figure S23C, Supporting Information).

Due to extremely rapid digestion rate, DTX‐S‐S‐TG prodrug could rapidly incorporate its digestion product (MG‐like prodrug) into small micelles completely and efficiently, facilitating its intestinal absorption. But, the bulky α‐substituent in the sn‐2 position strongly impaired the intestinal digestion of TG‐like DTX prodrugs and the incorporation into the small mixed micelles, probably decreasing their oral absorption potential.

#### 2.3.3 Stability of Prodrugs in Rat Plasma

The stability of the four prodrugs in plasma was investigated and the prodrugs appeared to be extremely stable with no degradation of the TG‐like prodrugs within 24 h (data are not shown), probably due to the strong hydrophobicity of the triglycerides. Lipoprotein lipase (LPL), bound to the luminal surface of capillary endothelial cells and only active under a physiological environment, is responsible for the lipolysis of TG structures in plasma and is specially active in tumor sites.^18^ Rat plasma supplemented with high LPL activity in which TG prodrugs could be hydrolyzed to release MG prodrugs rapidly, was used in the in vitro plasma stability study. As shown in Figure 2E,F, all of the prodrugs were rapidly hydrolyzed into MG‐like prodrugs within 15 min. However, the linkage bonds showed a significant effect on DTX release in blood‐circulation. The DTX‐S‐S‐TG prodrug was converted totally into DTX after 4 h incubation with plasma. In comparison, DTX was released slowly from the DTX‐5C‐TG and DTX‐5C(Et)‐TG prodrug in rat plasma, with 63% and 81% of DTX released within 12 h. Moreover, there was only 10% of DTX released from DTX‐5C(Piv)‐TG after 12 h. The rank order of DTX release was as follows: DTX‐S‐S‐TG, DTX‐5C‐TG prodrug, DTX‐5C(Et)‐TG prodrug, and DTX‐5C(Piv)‐TG prodrug. With bulky α substituents in the linkers, the DTX‐5C(Et)‐TG and DTX‐5C(Piv)‐TG prodrugs were more sterically inaccessible to the ester enzyme, resulting in low DTX release.

### 2.4 Reduction‐Sensitive Release of DTX‐S‐S‐TG Prodrug

#### 2.4.1 In Vitro Drug Release in the Presence of Dithiothreitol (DTT)

**Figure 3:**
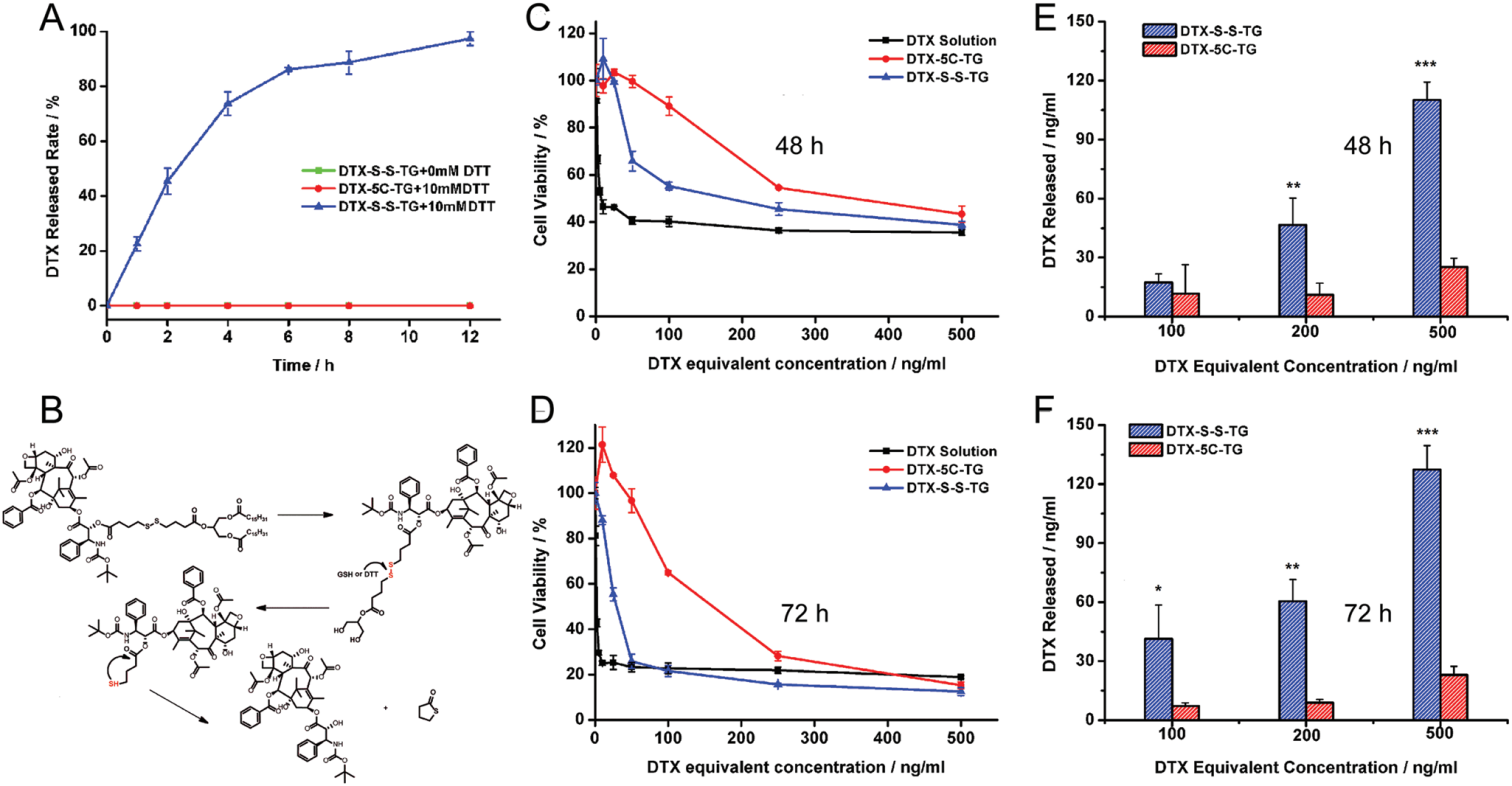
Reduction‐sensitive release of DTX‐S‐S‐TG prodrug. A) DTX release from TG prodrugs with or without DTT. B) The DTX release mechanisms of DTX‐S‐S‐TG prodrug. Cell viability of 4T1 cells after treated with various concentrations of DTX solution, DTX‐S‐S‐TG prodrug, and DTX‐5C‐TG prodrug for C) 48 h, D) 72 h. DTX released from prodrugs after incubation with 4T1 cells for E) 48 h, F) 72 h (data are represented as mean ± SD, *n* = 3, *P < 0.05, **P < 0.01, ***P < 0.001, the inset *P*‐values indicate the significance between groups).

The concentration of glutathione (GSH) in tumor cells is higher than that in normal cells, leading to a reduction intracellular microenvironment. To investigate the reduction‐sensitivity of DTX‐S‐S‐TG prodrug, DTT, a prevailing substitute of GSH, was used to simulate the reduction environment in the in vitro drug release study. As shown in **Figure** **3**A, the DTX‐S‐S‐TG prodrug released DTX rapidly in the presence of DTT, but there was no release of DTX in the absence of DTT. In addition, the reduction‐nonsensitive ester‐type prodrugs showed no release of DTX in response to DTT. These results indicated that the DTX‐S‐S‐TG prodrug could be rapidly specifically converted into the active parent drug in tumoral intracellular high reduction condition.

#### 2.4.2 In Vitro Cytotoxicity Assay and Drug Release in Tumor Cells

The in vitro cytotoxicity of the DTX‐S‐S‐TG prodrug, DTX‐5C‐TG prodrug, and the DTX solution were evaluated by MTT assays in 4T1 cells, KB cells, and L02 cells, respectively. As shown in Figure 3C,D; Figure S24 and Table S1, Supporting Information, the cytotoxicity of the DTX‐S‐S‐TG prodrug was higher than that of the DTX‐5C‐TG prodrug but lower than that of DTX solution due to delayed release of DTX in 4T1 cells and KB cells. The enhanced tumor cell killing effect of the DTX‐S‐S‐TG prodrug compared with the DTX‐5C‐TG may be attributed to faster drug release in tumor cells. More important, as shown in Figure S25, Supporting Information, there was no obvious cytotoxicity to L02 cells of the DTX‐S‐S‐TG, but relatively high cytotoxicity to 4T1 cells with the same DTX equivalent concentration, probably due to delayed DTX release in normal cells.

To prove above hypothesis, we further measured the DTX release from the prodrug after incubation with 4T1 cells for 48 and 72 h. As shown in Figure 3E,F, the amount of DTX released from the DTX‐S‐S‐TG prodrug was much higher than that released from the DTX‐5C‐TG prodrug at both 48 and 72 h. Then the intracellular GSH concentration in both normal cells and tumor cells were assayed. As shown in Figure S26, Supporting Information, the concentration of GSH in 4T1 cells and KB cells were significant higher than that in L02 cells. Similar results were also reported in the previous study of our group.^19^

To sum up, the DTX‐S‐S‐TG prodrug was triggered to rapidly release DTX in the presence of high levels of intracellular GSH in tumor cells whereas remained stable in normal cells. However, the nonsensitive DTX‐5C‐TG prodrug could only be hydrolyzed by nonspecific esterase, leading to a slow drug release rate and poor cytotoxicity. These results agreed well with the in vitro release study, confirming the tumor cell‐specific release property of DTX‐S‐S‐TG prodrug. The drug release mechanism could be summarized in Figure 3B.

### 2.5 In Situ Perfusion Study

The intestinal absorption of the TG prodrugs was evaluated using an in situ recirculating perfusion study. The absorption rate *K*_a_ of the TG prodrugs and the DTX formulations were shown in Figure S26, Supporting Information. The *K*_a_ values of all of the TG prodrugs and the DTX emulsion were higher than that of the DTX solution. The *K*_a_ values of the DTX‐S‐S‐TG prodrug, DTX‐5C‐TG prodrug, DTX‐5C(Et)‐TG prodrug, DTX‐5C(Piv)‐TG prodrug, and the DTX emulsion showed 5.13‐, 1.91‐, 1.73‐, 1.15‐, 1.72‐fold increases in comparison with the DTX solution, respectively. When the lipase inhibitor orlistat was coadministered, the *K*_a_ values of the four prodrugs were decreased significantly, in particular for DTX‐S‐S‐TG prodrug but had no effect for the DTX solution. Therefore, intestinal digestion mediated by lipase plays an important role in the intestinal absorption of TG prodrugs. The *K*_a_ values of DTX‐5C‐TG prodrug and DTX‐5C(Et)‐TG prodrug group were improved in comparison with DTX solution group, but lower than that of the DTX‐S‐S‐TG prodrug. The *K*_a_ value of the DTX‐5C(Piv)‐TG prodrug and the DTX solution was the lowest. These results could be explained by two reasons. First, high hydrophobic TG prodrugs have difficulties in crossing the UWL. In contrast, degraded MG prodrugs could form colloidal structures such as micelles that are more able to cross the UWL. The ability of digestion of the four prodrugs was quite different based on the stability study in bile‐pancreatic juice. Second, the digestion products sn‐2 monoglyceride and fatty acid could be absorbed into enterocytes via passive transport.^20^

### 2.6 In Vivo Oral Pharmacokinetic Study

**Figure 4:**
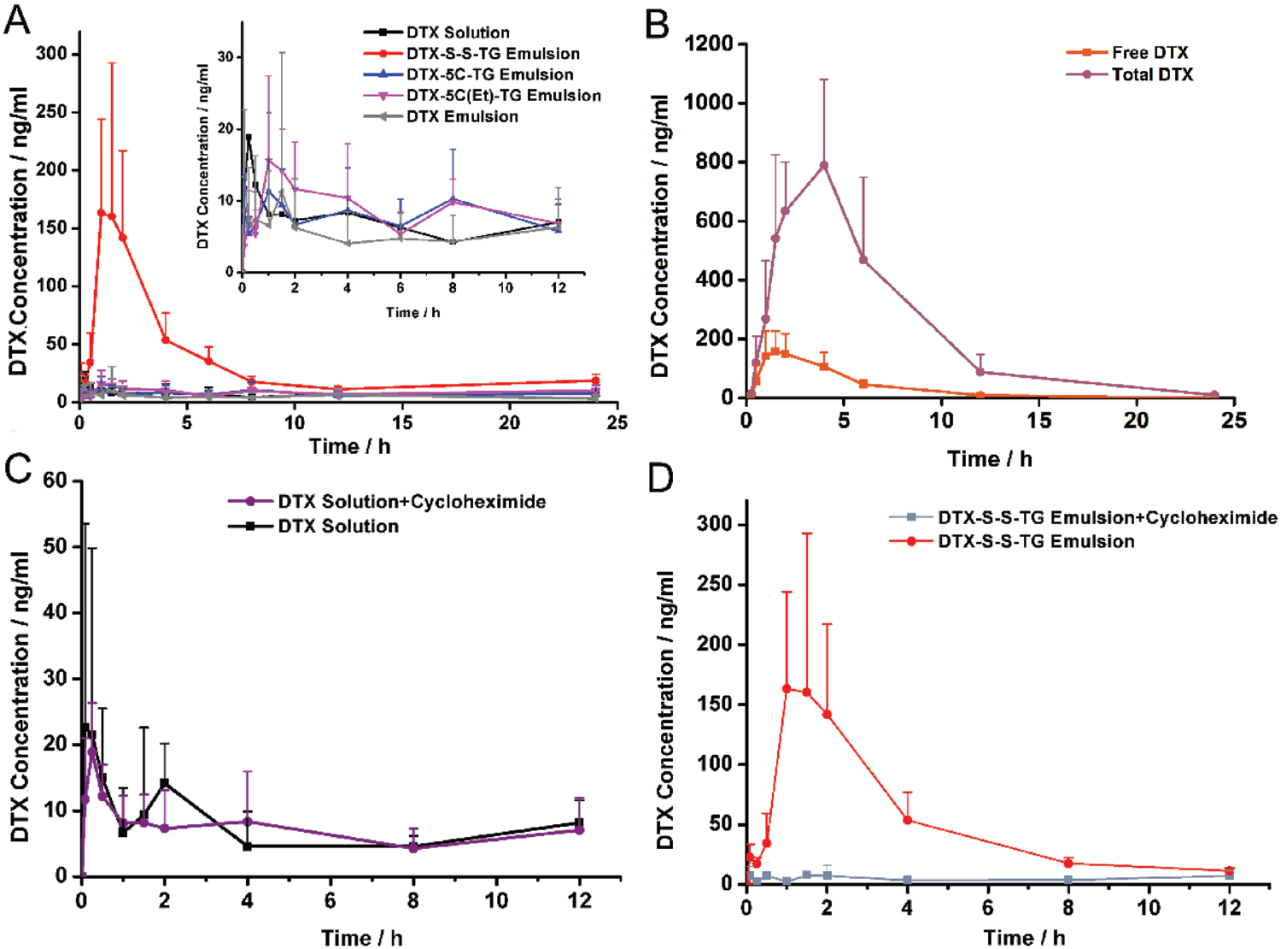
A) Mean plasma concentration‐time profiles of DTX solution, DTX‐5C(Et)‐TG emulsion, DTX‐5C‐TG emulsion, DTX emulsion, and DTX‐S‐S‐TG emulsion after oral administration in rats (data were represented as mean ± SD, *n* = 5). B) Mean plasma concentration−time profiles of the free and total DTX after oral administration of DTX‐S‐S‐TG emulsion (data were represented as mean ± SD, *n* = 4). C,D) Mean plasma concentration−time profiles of DTX solution and DTX‐S‐S‐TG emulsion after oral administration to nontreated rats, to cycloheximide‐treated rats 1 h before administration (data are represented as mean ± SD, *n* = 5 or 4).

The plasma concentration of DTX versus time profile after oral administration of TG‐like prodrug emulsions or DTX formulations were shown in **Figure** **4**A,B and the corresponding pharmacokinetic parameters were presented in **Table** **2**. It could be seen that oral administration of the DTX‐S‐S‐TG prodrug emulsion, DTX‐5C(Et)‐TG prodrug emulsion, and DTX‐5C‐TG prodrug emulsion showed 4.71‐, 1.48‐, and 1.05‐fold in DTX AUC_(0–24 h)_ compared with the DTX solution. Additionally, there was the largest increase in the *C*_max_ for the DTX‐S‐S‐TG prodrug emulsion (up to 235.5 ± 120.3 ng mL^−1^), ≈11.2‐fold higher than the DTX solution (21.00 ± 6.89 ng mL^−1^). In addition, the oral relative bioavailability (*F*_rel_) of the DTX‐S‐S‐TG prodrug emulsion, DTX‐5C(Et)‐TG prodrug emulsion and DTX‐5C‐TG prodrug emulsion were 470.7%, 148.3%, and 105.3%. Moreover, the oral absolute bioavailabilities (*F*_ab_) of these three prodrugs were 44.3%, 14%, and 9.9%, in sharp contrast to the 9.4% of the DTX solution. The DTX emulsion had a poor oral absorption with *F*_abs_% being as low as 6.7%. The plasma concentration of DTX of the DTX‐5C(Piv)‐TG prodrug emulsion group were not illustrated because the concentration of DTX was below the limit of quantitation. These results were in a good agreement with the in vitro studies. With good intestinal digestion and a stimuli‐responsive linkage, the oral bioavailability of DTX in the DTX‐S‐S‐TG prodrug group was the highest. In contrast, inefficient intestinal digestion of the DTX‐5C(Piv)‐TG prodrug lead to a limited intestinal absorption and very low oral bioavailability.

**Table 2:** Pharmacokinetic parameters of DTX solution, DTX‐5C(Et)‐TG emulsion, DTX‐5C‐TG emulsion, DTX emulsion, and DTX‐S‐S‐TG emulsion after oral administration in rats (mean ± S.D. *n* = 5)

Formulations	*t*_max_ [h]	*C*_max_ [ng mL^−1^]	*t*_1/2_ [h]	AUC_(0−24 h)_	*F*_rel_ [%]	*F*_ab_ [%]
DTX Solution	0.5 ± 0.6	21.0 ± 6.9	11.9 ± 8.5	167.8 ± 125.1	100.0	9.4
DTX emulsion	0.5 ± 0.6	18.5 ± 17.2	12.4 ± 8.5	119.9 ± 111.7	71.4	6.7
DTX‐5C‐TG	2.8 ± 3.3	19.0 ± 4.9	12.5 ± 4.1	176.8 ± 101.3	105.3	9.9
DTX‐5C(Et)‐TG	6.0 ± 10.0	25.0 ± 11.8	25.2 ± 20.3	248.8 ± 111.4	148.3	14.0
DTX‐S‐S‐TG	1.7 ± 0.5	235.5 ± 120.3	8.7 ± 9.0	789.9 ± 221.2	470.7	44.3

Following absorption into enterocyte, the 2‐MG prodrug was re‐esterified with endogenous or exogenous long‐chain fatty acids, resulting in the formation of a series of new TG derivatives of DTX, which made it impossible to directly determine the plasma concentration of the native prodrugs. Hence, the plasma samples were treated to liberate DTX from the DTX‐S‐S‐TG prodrug. Then the total DTX and the free DTX was quantified respectively. As shown in Figure 4B, the *C*_max_ (863.3 ± 188.5 ng mL^−1^) and the AUC_(0–24 h)_ (5542.7 ± 2368.3 µg L h^−1^) of total DTX were significantly higher than that of free DTX (179.6 ± 81.6 ng mL^−1^ and 788.2 ± 258.3 µg L h^−1^). The amount of DTX in its prodrug form was about 6.03‐fold to the released DTX, indicating that the majority of the DTX derivates were in its prodrug form. There are some reductants of low concentration such as glutathione in plasma, leading to partial drug release. However, the high hydrophobicity and disulfide bond protected the majority of prodrug from releasing in blood. According to the results of plasma stability and in vitro release, the complete DTX release occurred in the condition of a high level of LPL or in a strong reduction environment. This property ensured on site tumor‐bioactivation and made it a promising method to deliver drug into the target safely and effectively.

TG is absorbed mainly by the mesenteric lymph and oral absorption of TG mimetic prodrugs may follow the same pathway. To prove our hypothesis, an inhibitor of lymphatic transport, cycloheximide (3 mg kg^−1^), was used to block the chylomicron flow. As depicted in Figure 4C,D, the DTX plasma concentration after oral administration of the DTX‐S‐S‐TG prodrug emulsion was decreased significantly in cycloheximide‐treated rats. In contrast, there was little influence on the plasma concentration of DTX after oral administration of the DTX solution by cycloheximide pretreatment. These results confirmed that the TG‐like prodrugs were assembled into chylomicrons just like nature TG and prone to transport through lymphatic pathway, which avoided the first‐pass metabolism.

### 2.7 Absorption Mechanism and Biodistribution of TG‐Mimetic Prodrug

**Figure 5:**
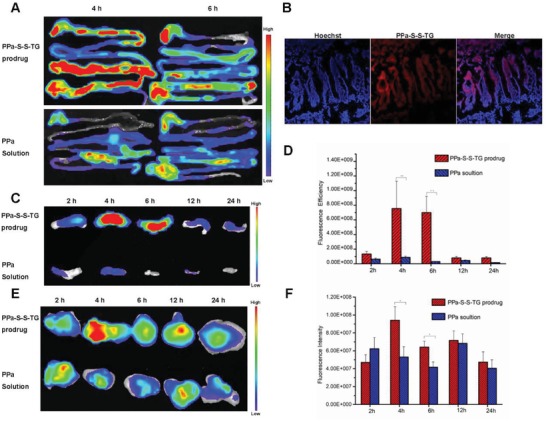
A) Ex vivo imaging of the distributions of the PPa‐S‐S‐TG and the PPa in GIT after 4 and 6 h of oral administration. B) CLSM observation of the intestinal tissue of the PPa‐S‐S‐TG group at 4 h. C) Ex vivo fluorescence images of the MLNs. D) Fluorescence intensities of the PPa‐S‐S‐TG and the PPa in MLNs. E) Ex vivo fluorescence images of the tumors. F) Fluorescence intensities of the PPa‐S‐S‐TG and the PPa in tumors.

To further illustrate the aborption mechanism of the TG‐like prodrug and its distrution in tumor, a TG‐mimetic dye, PPa‐S‐S‐TG was synthesized and the chemical structure was confirmed by mass spectra, ^1^H NMR,^13^C NMR, and infrared spectroscopy in Figures S15–S18, Supporting Information. The optical properties of PPa‐S‐S‐TG were also investigated and the results were shown as Figures S19 and S20, Supporting Information. The clogP of PPa‐S‐S‐TG was 18.45 and the simulated intestinal digestion result of the PPa‐S‐S‐TG was shown as Figure S28, Supporting Information, confirming the efficient intestinal digestion of the TG‐like dye, is identical to DTX‐S‐S‐TG. The reduction‐responsibility of the PPa‐S‐S‐TG was also verified as Figure S29, Supporting Information. After oral administration of the PPa‐S‐S‐TG emulsion and the PPa solution, GITs, mesenteric lymph nodes (MLNs) and tumors in the tumor‐bearing mice were taken out for ex vivo fluorescence image. As shown in **Figure** **5**A, the PPa‐S‐S‐TG were distributed widely in the GIT at 4 h especially in jejunum and ileum, while the fluorescence intensity of the PPa solution was much weaker. Until 6 h, there were still considerable amount of fluorescence response thoughout the GIT in the PPa‐S‐S‐TG group; however, the signal of the PPa solution group remained low. The intestinal absorption of the PPa‐S‐S‐TG was also visualized using the confocal laser scanning microscope (CLSM) and the results were demonstrated in Figure 5B. The PPa‐S‐S‐TG were observed within the intestinal villi instead of distribution at the surface of the villi 4 h after oral administration. Lacteals, lymphatic capillaries that are buried in the intestinal villi, is the gate by which chylomicron enter into lymph system.^21^ The chylomicron containing PPa‐S‐S‐TG may be transported into lacteals after being exocytosed from enterocyte according to the Figure 5B. The lymph transport was further validated by MLNs distribution. As shown in Figure 5C,D, fairly large amounts of the PPa‐S‐S‐TG accumulated in the MLNs, while rare PPa was observed in the PPa solution group. Together with the above results, the TG‐like dye or prodrug could maintain natural TG properties that go through the process of digestion, absorption into enterocyte, reesterification and assemble into chylomicron, transport to lymph system and then to circulation. The process could avoid the first‐pass effect so that improve the oral absorption of TG‐like prodrugs.

The biodistribution in tumor was also evaluated. As shown in Figure 5E,F, at 4 h, the fluorescence signal in tumor of the PPa‐S‐S‐TG emulsion group reach in its strongest, significantly higer than that of the PPa solution group. And the accumulation of the TG‐like dye in tumors were higher than the PPa solution group at almost all the timepoints. The high accumulation in tumor of the TG‐like dye may be due to the improved oral absorption, and the TG‐like prodrugs may act in the same manner.

### 2.8 In Vivo Antitumor Effect

**Figure 6:**
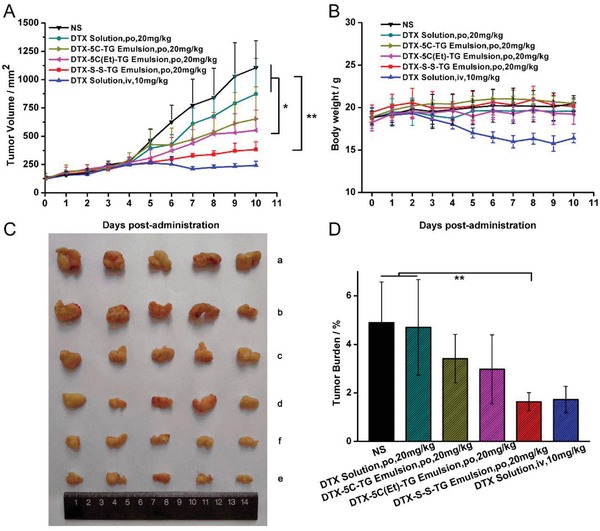
A) In vivo antitumor activity of prodrug against 4T1 xenograft tumors. B) Body weight change in each group. C) Images of tumors after the last treatment: (a) NS; (b) DTX Solution, po, 20 mg kg^−1^; (c) DTX‐5C‐TG Prodrug Emulsion, po, 20 mg kg^−1^; (d) DTX‐5C(Et)‐TG Prodrug Emulsion, po, 20 mg kg^−1^; and (e) DTX‐S‐S‐TG Prodrug Emulsion, po, 20 mg kg^−1^; (f) DTX Solution, iv, 10 mg kg^−1^. D) Tumor burden efficiency of each group (data are represented as mean ± SD, *n* = 5, *P < 0.05, **P < 0.01, The inset *P*‐values indicate the significance between groups).

The antitumor efficiency of TG prodrugs was evaluated in 4T1 tumor bearing mice. The tumor growth curve and physical photo of tumor were shown in **Figure** **6**A,C. The weight change curve and tumor burden efficiency were shown in Figure 6B,D. the DTX‐S‐S‐TG, DTX‐5C‐TG, and DTX‐5C(Et)‐TG showed effective tumor inhibition, especially for the DTX‐S‐S‐TG prodrug. The tumor burden efficiency of the DTX‐S‐S‐TG prodrug (po, 20 mg kg^−1^) was comparable to that of the DTX solution (i.v., 10 mg kg^−1^). Although the DTX solution (i.v.) demonstrated good tumor inhibition, the body weight of mice declined dramatically compared with the other groups. Additionally, H&E‐stained pathological section results were shown in Figure S30, Supporting Information. As for the liver, a mass of aggregating deep‐dyed big nucleolus cells were observed in the saline and the DTX solution groups (po, 20 mg kg^−1^), but no observation for the DTX‐S‐S‐TG prodrug group and the DTX solution (i.v.) group, which also showed a widespread karyopyknosis and nuclear fragmentation of the tumor cells. There was no obvious histological damage in the other organs in all of the groups. These results demonstrated that the DTX‐S‐S‐TG prodrug, with good oral absorption and on‐site tumor bioactivation, was outstanding in tumor growth inhibition without any severe side effects.

### 2.9 Gastrointestinal Toxicity Evaluation

**Figure 7:**
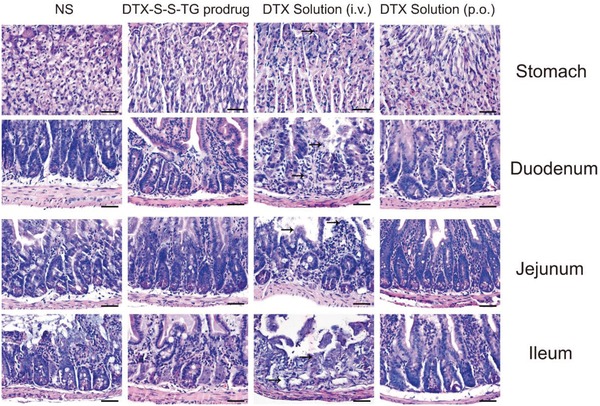
H&E stained tissue sections from mice after continuous oral administration with saline, DTX (20 mg kg^−1^), DTX‐S‐S‐TG prodrug emulsion (20 mg kg^−1^ equivalent to DTX) or intravenous injection with DTX solution (10 mg kg^−1^) for 5 d. Tissues were harvested from stomach, duodenum, jejunum and ileum.

High oral exposure to DTX may cause severe gastrointestinal tract toxicity, so H&E‐stained pathologic examination of the stomach, duodenum, jejunum, and ileum were carried out after continuous oral administration with the DTX solution (20 mg kg^−1^), DTX‐S‐S‐TG prodrug emulsion (20 mg kg^−1^ equivalent to DTX) or intravenous injection with DTX solution (10 mg kg^−1^) for 5 d. As shown in **Figure** **7**, there were different levels of inflammation in all parts of the gastrointestinal tract and severe cell apoptosis in the jejunum and ileum of the DTX solution (i.v.) group, probably due to the wide distribution and high concentration of DTX in the intestine. Oral administration of the DTX solution also caused some mucosa injury to the ileum but less severe GI toxicity than the DTX solution (i.v.), probably because the DTX solution has a very low oral bioavailability and elicits low concentration of DTX in enterocytes. In contrast, pathologic examination of gastrointestinal tract of the DTX‐S‐S‐TG group showed nearly no GI toxicity, very similar to saline group, without significant mucosa and cell injury. The underlying reason was that active DTX is not released from DTX‐S‐S‐TG during oral intestinal transport. This outstanding property indicates development of TG‐like DTX prodrug oral product is promising. In addition, hepatic and renal function analyses were performed (Figure S31, Supporting Information). AST, ALT, and CR in the DTX solution group were higher than in the other groups and no significant change was observed in the prodrug group, confirming the good safety of the TG‐like prodrugs.

## 3 Conclusions

A triglyceride‐mimetic prodrug strategy has been implemented to facilitate oral absorption of DTX by simulating the lymph transport of natural TG which avoids the first‐pass metabolism. The lymph transport mechanism was verified by the quite strong accumulation of the PPa‐S‐S‐TG in MLNs. Moreover, DTX‐S‐S‐TG exhibited an excellent tumor suppression effect and showed significantly less GI toxicity compared with oral and intravenous DTX solution. The development of TG‐like chemotherapeutical oral prodrugs has two important determinants: efficient intestinal digestion and on‐site tumor bioactivation. They can both be met by avoiding bulky branch at the α position to the glyceride ester and by introducing a reduction‐responsive cleavable linkage bond. This is the first time that the role of intestinal digestion in oral delivery of TG‐like prodrugs has been proposed and the reduction‐sensitive disulfide bond could achieve good safety and on‐demand tumor intracellular bioactivation. Such a bioinspired TG‐mimetic prodrug strategy gives new insight into the development of good oral chemotherapeutical products.

## Conflict of Interest

The authors declare no conflict of interest.

## Supporting information

Supporting Information

Supplemental Excel 1

Supplemental Excel 2

Supplemental Excel 3

Supplemental Excel 4

Supplemental Excel 5

Supplemental Excel 6

Supplemental Excel 7

Supplemental Excel 8

Supplemental Excel 9

Supplemental Excel 10

Supplemental Excel 11
