# Supplementary material for: Efficient Intestinal Digestion and On Site Tumor‐Bioactivation are the Two Important Determinants for Chylomicron‐Mediated Lymph‐Targeting Triglyceride‐Mimetic Docetaxel Oral Prodrugs
Source: Adv Sci (Weinh). 2019 Oct 25;6(24):1901810. doi: 10.1002/advs.201901810 (PMC6918103; doi:10.1002/advs.201901810)
Supplement: Supplementary file 1 — Supporting Information [file ADVS-6-1901810-s001.pdf]

Copyright WILEY-VCH Verlag GmbH & Co. KGaA, 69469 Weinheim, Germany, 2019.

ADVANCED SCIENCE
Open Access

# Supporting Information

for *Adv. Sci.,* DOI: 10.1002/advs.201901810

# Efficient Intestinal Digestion and On Site Tumor-Bioactivation are the Two Important Determinants for Chylomicron-Mediated Lymph-Targeting Triglyceride-Mimetic Docetaxel Oral Prodrugs

*Chutong Tian, Jingjing Guo, Gang Wang, Bingjun Sun, Kexin Na, Xuanbo Zhang, Zhuangyan Xu, Maosheng Cheng, Zhonggui He,* and Jin Sun**

**Efficient Intestinal Digestion and On Site Tumor-bioactivation are the Two Important Determinants for Chylomicron-mediated Lymph-targeting Triglyceride-mimetic Docetaxel Oral Prodrugs**

*Chutong Tian, Jingjing Guo, Gang Wang, Bingjun Sun, Kexin Na, Xuanbo Zhang, Zhuangyan Xu, Zhonggui He * and Jin Sun **

C. Tian, J. Guo, Dr. B. Sun, K. Na, X. Zhang, Z. X

Department of Pharmaceutics, Wuya College of Innovation, Shenyang Pharmaceutical University, No. 103, Wenhua Road, Shenyang, 110016, China

Dr. G. Wang

School of Pharmacy, Guang xi university of chinese medicine,Wuhe Rode, Nangning, 530200, China

Prof. Z. He

Department of Pharmaceutics, Wuya College of Innovation, Shenyang Pharmaceutical University, Shenyang 110016, PR China;

E-mail address: hezhonggui@vip.163.com;

Prof. J. Sun

Municipal Key Laboratory of Biopharmaceutics, Wuya College of Innovation, Shenyang Pharmaceutical University, Shenyang 110016, PR China

E-mail address: sunjin@syphu.edu.cn

## Experimental Section

### *Materials*

DTX was purchased from Nanjing Jingzhu Biological Engineering Co., Ltd., China. Egg yolk lecithin was obtained from Shanghai Advanced Vehicle Technology L.T.D. Co. Sodium deoxycholate, olive oil, 4-dimethylaminopyridine (DMAP), 1-Ethyl-3-(3-dimethylaminopropyl) carbodiimide hydrochloride (EDCI), 4,4'-dithiodibutyric acid and dithiothreitol (DTT) were obtained from Aladdin Industrial Corporation (Shanghai, China). Orlistat, 3-(4,5-dimethylthiazol-2-yl)-2,5-diphenyl tetrazolium bromide (MTT), TCEP ( Tris(2-carboxyethyl)phosphine ) and cycloheximide were obtained from Dalian Meilun biotechnology Co., Ltd., China. Lipoprotein lipase was obtained from Sigma-Aldrich, MO, USA. All other solvents and reagents used were of analytical quality.

### *Design and synthesis of the triglyceride mimetic prodrug and the triglyceride mimetic dye*

Four prodrugs of DTX: DTX-5C-TG, DTX-5C(Et)-TG, DTX-5C(Piv)-TG and DTX-S-S-TG and the triglyceride-like dye PPa-S-S-TG were designed and synthesized and their chemical structures are shown in Fig. S1 and S2. The synthesis approach is summarized in Scheme S1 and S2 and the chemical structures of the prodrugs were confirmed by high resolution mass spectra, $^{1}H$ NMR, $^{13}C$ NMR and infrared spectroscopy.

*Synthesis of prodrugs DTX-5C(Et)-TG and DTX-5C(Piv)-TG* L-glutamic acid γ-benzyl ester (**S1**, 1.5 g, 6.33 mmol) was dissolved in a mixed solvent (water : HAc 1:1) and was stirred at 0°C for 20 min. $NaNO_2$ (3.45 g, 50 mmol) was dissolved in 5 mL distilled water and dropped into the solution above and stirred for a further hour at 0°C. Then, the reaction temperature was turned up to 25°C and stirred overnight. The reaction mixture was quenched with ethyl acetate and the organic phase was dried ($Na_2SO_4$) and concentrated to get the crude product of compound **1**, which was redissolved in dry $CH_2Cl_2$ (30 mL) containing DMAP (100 mg, 0.8 mmol). Acetic anhydride (0.6 g, 5.88 mmol) or pivaloyl chloride (770 μL, 6.28 mmol) was added and reacted at 25°C

overnight. The reaction mixture was washed with 1 N HCl and quenched with $CH_2Cl_2$ and then the organic phase was dried ($Na_2SO_4$) and concentrated to get a crude product of compound **2** or **3**. Silica gel chromatography was used for purification (10:1 petroleum ether/ethyl acetate). A mixture of 1,3-DG (900 mg, 1.58 mmol, synthesized according to a previously reported method [1]), DMAP (200 mg, 1.60 mmol), EDCI (700 mg, 3.66 mmol), and corresponding amount of compound **2** or compound **3** was dissolved in dry $CH_2Cl_2$ (30 mL) and stirred at 25°C for 48 h. The reaction mixture was concentrated under reduced pressure. Silica gel chromatography (15:1 n-hexane/ethyl acetate) was used to give the pure intermediate **4** or **5**. Compound **4** or **5** (600 mg) was dissolved in 20 mL THF, Pd/C (60 mg, 10%) was added slowly and the reaction was stirred under an $H_2$ atmosphere at 20°C for 4 h and then filtered. The THF was evaporated to get compound **6** or **7**. A mixture of DTX (**$S_2$**, 803 mg, 1 mmol), DMAP (45 mg, 0.36 mmol), EDCI (175 mg, 0.91 mmol), and the corresponding amount of compound **6** or **7** in dry $CH_2Cl_2$ (30 mL) was stirred at 25°C for 48 h, the $CH_2Cl_2$ was evaporated and the crude product was purified by silica gel chromatography (2:1 n-hexane/ethyl acetate) to give the pure product of DTX-5C(Et)-TG (compound **8**) or DTX-5C(Piv)-TG (compound **9**). DTX-5C(Et)-TG $^1$H NMR (400 MHz, $CDCl_3$, ppm) δ 8.12 (d, *J* = 7.5 Hz, 2H), 7.61 (t, *J* =14.1 Hz, 1H), 7.51 (t, *J* = 7.6 Hz, 2H), 7.43 – 7.24 (m, 5H), 6.23 (s, 1H), 5.69 (d, 1H), 5.49 (s, 1H), 5.36 (s, 1H), 5.31 (m, 1H), 5.21 (s, 1H), 4.99 (m, 1H), 4.97 (m, 1H) , 4.33 (d, 1H),4.20-4.18 (m, 4H), 3.94 (d, J = 6.8 Hz, 1H), 2.57 (m, 2H), 2.44 (s, 3H), 2.31 (q, 4H),2.28 (dt, 2H), 2.15 (m, 1H), 2.10 (s, 3H) 1.95 (s, 3H), 1.87 (m, 1H), 1.76 (s, 3H), 1.59 (m, 4H), 1.33(s, 9H) 1.30-1.24 (m , 48H), 1.23 (s, 3H), 1.13 (s, 3H), 0.88 (t, *J* = 6.8 Hz, 6H). $^{13}$C NMR (100 MHz, $CDCl_3$) δ 211.56, 173.36, 171.26, 169.99, 169.72, 168.77, 167.96, 167.09, 135.59, 133.65, 130.21, 129.25, 128.90, 128.72, 128.20, 126.35, 84.19, 81.03, 80.36, 78.93, 77.34, 77.23, 77.03, 76.71, 74.98, 74.61, 74.53, 71.96, 71.88, 70.72, 70.43, 61.83, 61.67, 57.53, 46.51, 43.10, 36.98, 35.53, 33.97, 33.95, 31.93, 29.71, 29.67, 29.64, 29.50, 29.37, 29.29, 29.15, 28.96, 28.16, 26.34, 25.68, 24.82, 22.70, 20.84, 20.43, 14.24, 14.13, 9.96. ESI-HRMS: Calcd. For $C_{85}H_{127}NNaO_{23}$ [M+Na$^+$] 1552.8742 found 1552.8691.

DTX-5C(Piv)-TG $^1$H NMR (400 MHz, $CDCl_3$, ppm) δ 8.05 (d, *J* = 7.4 Hz, 2H), 7.53

(dd, *J* = 14.1 Hz, 1H), 7.44 (t, *J* = 7.6 Hz, 2H), 7.39 – 7.17 (m, 5H), 6.16 (s, 1H), 5.62 (d, 1H), 5.51 (s, 1H), 5.44 (d, *J* = 14.5 Hz, 1H), 5.29 (m, *J* = 14.5 Hz, 1H), 5.15 (s, 1H), 4.92 (m, 1H), 4.90 (m, 1H) , 4.26 (d, 1H), 4.17-4.06 (m, 4H), 3.87 (d, *J* =7.1 Hz, 1H), 2.50 (m, 2H), 2.37 (s, 3H), 2.24 (q, 4H), 2.07 (dt, 2H), 2.02 (m, 1H), 1.88 (s, 3H), 1.79 (m, 1H), 1.69 (s, 3H), 1.53 (m, 4H), 1.26 (s, 9H) 1.20-1.18 (m, 48H), 1.15 (s, 3H), 1.14 (m, 9H), 1.06 (s, 3H), 0.83 (t, *J* = 6.8 Hz, 6H). $^{13}$C NMR (100 MHz, $CDCl_3$) δ 211.56, 173.36, 171.26, 169.99, 169.72, 168.77, 167.96, 167.09, 135.59, 133.65, 130.21, 129.25, 128.90, 128.72, 128.20, 126.35, 84.19, 81.03, 80.36, 78.93, 77.34, 77.23, 77.03, 76.71, 74.98, 74.61, 74.53, 71.96, 71.88, 70.72, 70.43, 61.83, 61.67, 57.53, 46.51, 43.10, 36.98, 35.53, 33.97, 33.95, 31.93, 29.71, 29.67, 29.64, 29.50, 29.37, 29.29, 29.15, 28.96, 28.16, 26.34, 25.68, 24.82, 22.70, 20.84, 20.43, 14.24, 14.13, 9.96. ESI-HRMS: Calcd. For $C_{88}H_{134}NO_{23}$ [M+H$^+$] 1572.9291 found 1572.9341.

*Synthesis of prodrug DTX-5C-TG* 1.3-DG (500 mg, 0.88 mmol), glutaric anhydride (200 mg, 1.75 mmol) and DMAP (104 mg, 0.91 mmol) were dissolved in a mixed solvent ($CH_2Cl_2$/THF/pyridine 1:1:1) and reacted at 25 ℃ for 48 h. The reaction mixture was washed with 0.1 N HCl and quenched with $CH_2Cl_2$ and then the organic phase was dried ($Na_2SO_4$) and concentrated to get the crude product of compound **10**. Silica gel chromatography was used for purification (15:1 petroleum ether/ethyl acetate). A mixture of DTX (**S2**, 803 mg, 1 mmol), DMAP (45 mg, 0.36 mmol), EDCI (175 mg, 0.91 mmol), and compound **10** (560 mg, 0.83 mmol) in dry $CH_2Cl_2$ (30 mL) were stirred at 25℃ for 48 h, the $CH_2Cl_2$ was evaporated and the crude product was purified by silica gel chromatography (5:1 n-hexane/ethyl acetate) to give the pure product of DTX-5C-TG (compound **11**). $^{1}$H NMR (400 MHz, $CDCl_3$, ppm) δ 8.12 (d, J = 7.5 Hz, 2H), 7.61 (t, J = 7.5 Hz, 1H), 7.51 (t, J = 7.6 Hz, 2H), 7.43 – 7.24 (m, 5H), 6.25 (s, 1H), 5.70 (d, 1H), 5.50 (s, 1H), 5.37 (s, 1H), 5.31 (m, 1H), 5.21 (s, 1H), 4.97 (m, 1H), 4.33 (d, 1H), 4.20-4.17 (m, 4H), 3.94 (d, J = 6.9 Hz, 1H), 2.59 (m, 1H), 2.45 (s, 3H), 2.38 (m, 1H), 2.32 (dt, 2H), 2.31 (q, 4H), 2.18 (m, 1H), 1.96 (s, 3H), 1.88 (m, 2H), 1.76 (s, 3H), 1.59 (m, 4H), 1.33 (s, 9H) 1.30-1.24 (m , 48H), 1.23 (s, 3H), 1.13 (s, 3H), 0.88 (t, J = 6.8 Hz, 6H). $^{13}$C NMR (100 MHz, $CDCl_3$) δ 211.60, 173.37, 171.88,

171.80, 168.04, 167.09, 139.11, 135.56, 133.64, 130.21, 129.25, 128.88, 128.73, 128.17, 126.32, 84.18, 81.01, 80.35, 78.95, 77.34, 77.23, 77.02, 76.71, 74.99, 74.52, 74.41, 71.91, 69.29, 61.99, 57.56, 46.45, 43.11, 36.98, 35.59, 34.04, 32.76, 32.61, 31.93, 29.71, 29.67, 29.63, 29.49, 29.37, 29.28, 29.13, 28.16, 26.33, 24.86, 22.70, 20.90, 19.76, 14.23, 14.13, 9.96. ESI-HRMS: Calcd. For $C_{83}H_{126}NO_{21}$ [$M+H^+$] 1472.8811 found 1472.8816.

*Synthesis of prodrug DTX-S-S-TG* 4,4'-dithiodibutyric acid (2.1 g, 8.8 mmol) was dissolved in 9 mL acetic anhydride and stirred at 25℃ for 2 h and concentrated to get intermediate **12**, which was which was redissolved in dry $CH_2Cl_2$ (30 mL) containing 1.3-DG (500 mg, 0.88 mmol) and TEA (845 mg, 8.4 mmol) and reacted at 25℃ for 24 h. The reaction mixture was concentrated to get the crude product of compound **13**. Silica gel chromatography was used for purification (15:1 petroleum ether/ethyl acetate). A mixture of DTX (**S2**, 803 mg, 1 mmol), DMAP (45 mg, 0.36 mmol), EDCI (175 mg, 0.91 mmol), and compound **13** (759 mg, 0.83 mmol) in dry $CH_2Cl_2$ (30 mL) were stirred at 25℃ for 48 h, the $CH_2Cl_2$ was evaporated and the crude product was purified by silica gel chromatography (5:1 n-hexane/ethyl acetate) to give the pure product of DTX-S-S-TG (compound **14**). $^1$H NMR (400 MHz, $CDCl_3$, ppm) δ 8.12 (d, J = 7.5 Hz, 2H), 7.62 (t, J = 7.5 Hz, 1H), 7.55 (t, J = 7.6 Hz, 2H), 7.44 – 7.24 (m, 5H), 6.23 (s, 1H), 5.69 (d, 1H), 5.48 (s, 1H), 5.38 (s, 1H), 5.34 (m, 1H), 5.25 (s, 1H), 4.97 (m, 1H), 4.30 (d, 4H), 4.20-4.17 (m, 4H), 3.94 (d, J = 6.8 Hz, 1H), 2.70 (m, 2H), 2.58 (m, 4H), 2.47 (m, 2H), 2.42 (s, 3H), 2.31 (q, 4H), 2.18 (m, 1H), 1.96 (s, 3H), 1.85 (m, 2H), 1.76 (s, 3H), 1.59 (m, 4H), 1.31 (s, 9H) 1.30-1.24 (m, 48H), 1.23 (s, 3H), 1.13 (s, 3H), 0.88 (t, J = 6.8 Hz, 6H). $^{13}$C NMR (100 MHz, $CDCl_3$) δ 211.56, 173.36, 171.26, 169.99, 169.72, 168.77, 167.96, 167.09, 135.59, 133.65, 130.21, 129.25, 128.90, 128.72, 128.20, 126.35, 84.19, 81.03, 80.36, 78.93, 77.34, 77.23, 77.03, 76.71, 74.98, 74.61, 74.53, 71.96, 71.88, 70.72, 70.43, 61.83, 61.67, 57.53, 46.51, 43.10, 36.98, 35.53, 33.97, 33.95, 31.93, 29.71, 29.67, 29.64, 29.50, 29.37, 29.29, 29.15, 28.96, 28.16, 26.34, 25.68, 24.82, 22.70, 20.84, 20.43, 14.24, 14.13, 9.96. ESI-HRMS: Calcd. For $C_{86}H_{132}O_{21}S_2$ [$M+H^+$] 1578.8709 found 1578.8727.

*Synthesis of the TG-like dye PPa-S-S-TG* 3,3'-dithiodibutyric acid (735 mg, 3.5 mmol) was dissolved in 9 mL acetic anhydride and stirred at 25°C for 2 h and concentrated to get intermediate **15**, which was which was redissolved in dry $CH_2Cl_2$ (30 mL) containing 1.3-DG (200 mg, 0.35 mmol) and TEA (845 mg, 8.4 mmol) and reacted at 25°C for 24 h. The reaction mixture was concentrated to get the crude product of compound **16**. Silica gel chromatography was used for purification (10:1 petroleum ether/ethyl acetate). The pure compound **16** (155 mg, 0.20 mmol) was dissolved in 10 mL ethylene glycol and p-methyl benzenesulfonic acid ( 3 mg, 0.02 mmol) was added and stirred at 110°C for 4 h. The reaction mixture was concentrated to get the crude product of compound **17**. A mixture of PPa (**S3**, 100 mg, 0.19 mmol), DMAP (23 mg, 0.18 mmol), EDCI (87 mg, 0.45 mmol), and compound **17** (160 mg, 0.20 mmol) in dry $CH_2Cl_2$ (30 mL) were stirred at 25℃ for 48 h, the $CH_2Cl_2$ was evaporated and the crude product was purified by silica gel chromatography (8:1 n-hexane/ethyl acetate) to give the pure product of PPa-S-S-TG (compound **18**). $^1$H NMR (400 MHz, $CDCl_3$, ppm) δ 9.50, 9.39 (each s, each 1H), 8.56 (s, 1H), 8.02-7.95 (dd, 1H), 6.33 (d, 1H), 6.23 (d,1H), 5.30 (d, 1H), 5.19 (d,1H), 4.85 (m, 1H), 4.47 (m, 1H), 4.43-4.22 (m, 4H), 4.22-4.09 (m, 5H), 3.84 (q, 2H), 3.67 (s, 3H), 3.41 (s, 3H), 3.24 (s, 3H), 2.78-2.71 (m, 4H), 2.71-2.64 (m, 4H), 2.61-2.52 (m, 2H), 2.35-2.30 (m, 4H), 2.28-2.25 (m, 2H), 1.79 (d, 3H), 1.70 (t, 3H), 1.57 (m, 4H), 1.26 (m, 48H), 0.88 (t, 6H), 0.52 (br, 1H), -1.6 (br, 1H). $^{13}$C NMR (100 MHz, $CDCl_3$) δ 196.13, 173.37, 173.27, 172.81, 171.35, 170.73, 160.15, 155.28, 150.84, 149.02, 145.06, 141.57, 137.91, 136.24, 136.14, 135.92, 131.58, 129.24, 128.38, 122.59, 106.06, 104.16, 97.25, 93.02, 77.34, 77.23, 77.02, 76.70, 69.74, 69.62, 66.44, 62.41, 62.17, 61.92, 61.88, 61.11, 51.64, 49.99, 48.07, 34.06, 34.01, 33.91, 33.80, 33.18, 32.88, 32.80, 31.93, 30.85, 29.69, 29.65, 29.61, 29.49, 29.46, 29.36, 29.28, 29.25, 29.14, 29.10, 24.87, 24.83, 23.16, 22.69, 19.50, 17.44, 14.12, 12.12, 12.07, 11.26. ESI-HRMS: Calcd. For $C_{76}H_{113}N_4O_{11}S_2$ [M+H$^+$] 1321.7798 found 1321.7841.

***Optical properties of PPa-S-S-TG***

PPa and PPa-S-S-TG were dissolved in acetonitrile and the equivalent concentration of PPa were 5 μM. The ultraviolet and fluorescence spectras were then obtained by microplate spectrophotometer (Thermo Scientific, USA).

***Preparation of chylomicron-like emulsions***

The emulsions were formulated by sonication. In detail, 120 mg egg lecithin and 40 mg sodium deoxycholate were dispersed in 4 mL deionized water at 60°C. In addition, 400 mg olive oil containing 12 mg TG prodrug or 6 mg DTX was heated to the same temperature. Then, the oil was dropwise added into the aqueous phase under stirring. The resulting pre-emulsion was sonicated at 500 W for 10 min in an ice bath. The dispersion was then filtered through a 0.45-μm filter to remove larger particles.

***Characterization of chylomicron-like emulsions***

The size, polydispersity index (PDI) and zeta potential of the emulsion particles were checked by a Nano ZS Zetasizer instrument (Malvern, UK). The encapsulation efficiency (EE) of the emulsions was evaluated by gel permeation chromatography. In brief, the emulsion (0.1 mL) was carefully applied to a Sephadex G-50 column (diameter 0.7 cm, length 25 cm) and eluted with water to separate the emulsion particles from the unincorporated prodrug or DTX. The amount of encapsulated drug in the eluent and the total drug in the emulsion was measured after acetonitrile (ACN) disruption by HPLC at 230 nm. The EE(%) was determined according to the following formula:

$$\mathrm{EE}(\%) = \left(\frac{W_1}{W_2}\right) \times 100\%$$

where $W_1$ is the amount of encapsulated drug and $W_2$ is the amount of the total drug in the emulsion.

***Stability studies***

*Stability in simulated gastric fluid (SGF)*

SGF was prepared according to the previously reported procedure [2]. To investigate the physical stability of the chylomicron-like emulsions, 1 mL emulsion was mixed with 9 mL SGF and incubated at 37°C with gentle shaking. The emulsion was sampled at regular intervals to measure the particle size [3]. To determine the chemical stability of the prodrugs, 100 μL emulsion was incubated with 900 μL SGF at 37°C with gentle shaking. Samples (20 μL) of the incubation solution were taken at regular intervals and added to 180 μL ACN to analyze the concentration of the prodrug by HPLC.

*Simulated intestinal digestion*

Bile-pancreatic juice was obtained from the common bile duct of anesthetized rats, which contained bile as well as pancreatic juice as previously described [4]. Then, 20 μL of emulsion was incubated with 180 μL bile-pancreatic juice at 37°C with gentle shaking to simulate intestinal digestion. Samples (20 μL) of the incubation solution were taken at regular intervals (0, 0.08, 0.25, 0.5, 1, 2, 3 h) and added to 180 μL of ACN to analyze the concentration of the TG prodrugs and the monoglyceride derivatives of DTX (MG prodrugs) by HPLC. The solubility of prodrugs in aqueous phase were also determined. 20 μL of emulsion was incubated with 180 μL bile-pancreatic juice at 37°C with gentle shaking for 3h and 20 μL of the incubation solution were taken and added to 180 μL of ACN to analyze the concentration of the TG prodrugs and the monoglyceride derivatives of DTX (MG prodrugs) by HPLC. Then the samples were centrifuged at 4°C and 12000 rpm for 30 min. The samples separated into a pellet phase in the bottom, a clear aqueous phase (mix micelles phase) in the middle and a thin cream phase (oil phase) at the top. 20 μL of aqueous phase were collected and the concentration of the TG prodrugs and MG prodrugs were determined as above. The fraction of TG-like prodrugs and MG-like prodrugs partitioning in mix micelle phase (aqueous phase) relative to those in raw digesta were calculated to evaluate the solubility of the prodrugs. At the same time, the particle sizes of mix micelles were measured by Malvern laser particle size analyzer.

*Stability of the prodrugs in rat plasma*

In order to study the stability of linkers in circulation, TG prodrugs were incubated with rat plasma supplemented with lipoprotein lipase (LPL, 10000 IU/mL) in which TG prodrugs can be hydrolyzed into MG prodrugs rapidly. Thus, 500 μL of rat serum supplemented with LPL (10000 IU/mL) was added to 50 μL of emulsion and incubated at 37°C. At scheduled times (0, 0.25, 0.5, 1, 2, 4, 8, 12 h), 40 μL samples were taken and added to 160 μL of ACN for analysis. The concentration of TG, MG prodrugs and free DTX were determined by HPLC.

***Reduction-sensitive release of the DTX-S-S-TG prodrug***

*In vitro drug release in the presence of DTT*

To investigate the controlled release of the DTX-S-S-TG prodrug, phosphate buffer solution (pH 7.4) (containing 30% ethanol (v/v)) with or without dithiothreitol (DTT) was used as the release media. DTT is a substitute of glutathione (GSH) and we fixed its concentration at 10 mM in reduction-sensitive release media. Then, 200 μL of the emulsions (DTX-S-S-DTX prodrug emulsion or DTX-5C-TG prodrug emulsion) were sealed into dialysis bags and incubated in 20 mL release media (reduction-sensitive or reduction-insensitive) at 37°C. At 0 h, 1 h, 2 h, 4 h, 6 h, 8 h and 12 h, 1 mL media was sampled and filtered through a 0.22-μm filter while 1 mL of fresh release media was added the reaction to maintain the total volume. The concentration of released DTX was determined by HPLC.

***Cell culture***

4T1 cells were cultured in RPMI 1640 medium with 10% FBS and KB cells were cultured in high glucose DMEM medium with 10% FBS. All cells were maintained in a humidified atmosphere of 5% $CO_2$ at 37 °C. All the cell culture plastic product were obtained from Wuxi NEST Biotechnology Co., Ltd.

***In vitro cytotoxicity and drug release in tumor cell***

The cytotoxicity of the DTX solution, DTX-5C-TG and DTX-S-S-TG were investigated in 4T1, KB and L02 cells. In brief, cells were added to 96-wells at a density of 5000 cells per well and cultured for 24 h and then the medium was discarded. The cells were incubated with different concentrations of DTX solution or prodrug emulsions for 48 h or 72 h and cell viability was determined by MTT assay. The cells without any treatment were utilized as control (n=3 for each group).

To evaluate the free drug released from prodrugs after incubation with 4T1 cells, the cells were seeded into 24-wells at a density of 5000 cells per well and cultured for 24 h. Then, the cells were incubated with different concentration of prodrugs (DTX equivalent concentration 100, 200 and 500 ng/mL) for 48 h or 72 h. After incubation, the cells and media were collected and after sonication and centrifugation, the concentration of DTX was determined by UPLC-MS-MS.

***Detection of cellular GSH***

L02 cells, 4T1 cells and KB cells were seeded in 12-wells plates respectively at a

density of $10*10^5$ cells per well and cultured for 24h. The cells were washed by PBS for 3 times and then digested and counted. The cell pellet was resuspended by protein removal reagent M solution. The cellular GSH concentration were then assayed using Trace Glutathione Assay Kit (Njjcbio, Nanjing, China).

***Animal studies***

Sprague–Dawley (SD) rats (180-220g) and BalB/c mic (18-22g) were obtained from the Laboratory Animal Center of Shenyang Pharmaceutical University (Shenyang, Liaoning, China). All the animal experiments were performed according to the Guidelines for the Care and Use of Laboratory Animals approved by the Institutional Animal Ethical Care Committee (IAEC) of Shenyang Pharmaceutical University.

***In situ perfusion study***

To evaluate the intestinal permeability of the prodrugs, an in situ recirculating perfusion study was applied as previously described [5] with slight modifications. In detail, the prodrug emulsions were diluted to 30 μg/mL and the DTX solution and emulsion to 15 μg/mL using Krebs Ringer's (KR) buffer solution as the perfusate. Male rats were fasted for 12 h prior to the experiment and then were anesthetized with an i.p. injection of urethane (1.0 g/kg). The rats were placed under an infrared lamp to maintain a normal body temperature. The abdomen was opened along the midline with a 3~4-cm longitudinal incision. The inlet tube was inserted at upper duodenum and the outlet tube was inserted at the end of the ileum. After gentle washing with 37°C KR buffer, the intestinal segment was covered with cotton wetted by saline. Both the inlet and outlet tube were put into the perfusate, which was reserved in a graduated cylinder to form a loop and the intestinal segment was equilibrated with perfusate at a flow rate of 5 mL/min for 20 min. The perfusion experiment was started with a flow rate of 2.5 mL/min and lasted for 2 h. A 100μL sample was collected from the perfusate every 15 min and the same volume of blank KR buffer was added. The volume of the perfusate was recorded at the same time. Each sample was diluted with 300μL acetonitrile for concentration determination by HPLC. For the inhibition experiment, orlistat was given at a dosage of 240 mg/kg (i.g.) before the surgery. The dead volume was determined by air blowing using the peristaltic pump. The absorption rate $K_a$ was calculated as follows:

$$A_{tn}=C_{tn}\cdot(V_{tn}+V_d)+0.1\cdot\sum_{i-1}^{n-1} C_{ti}$$

$$\ln A_{tn}=\ln A_{t0}-K_a\cdot t$$

where $A_{tn}$ is the amount of prodrug or DTX at different times in the perfusate. $C_{tn}$ and $V_{tn}$ are the concentration and volume of the perfusate at different times respectively. $V_d$ is the dead volume.

***In vivo oral pharmacokinetic study***

Sprague-Dawley (SD) male rats (weighting 200 to 220 g) were used to study the oral absorption of TG prodrugs and DTX formulations. The rats were fasted for 12 h before the experiment and divided into 5 groups (n=5 each group) randomly: DTX solution (i.g., 10 mg/kg), DTX emulsion (i.g., 10 mg/kg), DTX prodrug emulsions (i.g., 10 mg/kg equivalent to DTX). A 0.5-mL blood sample was collected into an EDTA-coated tube at 0.08 h, 0.25 h, 0.5 h, 1 h, 2 h, 4 h, 6 h, 8 h, 12 h and 24 h after administration with formulations above. To achieve absolute bioavailability, 4 rats were given intravenously a DTX dose of 5 mg/kg. For the inhibition experiment, cycloheximide, an inhibitor of lipid lymphatic transport, was given by intraperitoneal injection at a dose of 3 mg/kg and the DTX-S-S-TG prodrug emulsion (i.g., 10 mg/kg equivalent to DTX) and DTX solution (i.g., 10 mg/kg) were administered orally an hour later. Blood samples (0.5 mL) were taken from the orbit at 0.08 h, 0.25 h, 0.5 h, 1 h, 2 h, 4 h, 6 h, 8 h and 12 h. All of the blood samples were centrifuged immediately at 5000 rpm for 10 min after being collected and the plasma obtained was stored at -20°C until analysis.

The DTX concentrations in the plasma of all test groups were determined by a validated UPLC-MS/MS method after protein precipitation with ACN using diazepam as an internal standard. To determinate the total DTX concentrations of the DTX-S-S-TG group, Tris(2-carboxyethyl)phosphine (TCEP) and LPL were added to destroy the structure of the prodrug. Briefly, 50 μL plasma samples were firstly added with 50μL LPL (5000IU/ml) and 50μL 1mM TCEP then vortex mixed for 5 min to release DTX, and the following steps were same as the free DTX method as described above. The pharmacokinetic parameters were acquired by DAS software. The relative bioavailability ($F_{rel}$) and absolute bioavailability ($F_{ab}$) were calculated as follows:

$$F_{rel} = AUC_{Emulsion} \cdot Dose_{Sol} / AUC_{Sol} / Dose_{Emulsion}$$

$$\boldsymbol{F_{ab} = AUC_{p.o.} \cdot Dose_{i.v.} / AUC_{i.v.} / Dose_{p.o.}}$$

***Absorption mechanism and biodistribution of TG-mimetic prodrug***

The PPa-S-S-TG was synthesized as scheme S2, the PPa-S-S-TG emulsion and its simulated intestinal digestion were implemented in accordance with the step of the TG-like DTX prodrug emulsion. Its reduction-responsibility was verified by incubation PBS with or without DTT. In brief, phosphate buffer solution (pH 7.4) (containing 30% ethanol (v/v)) with or without dithiothreitol (DTT) was used as the release media. The concentration of DTT was fixed at 10 mM. Then, 200 μL of the emulsions (PPa-S-S-TG) was incubated with 20 mL release media (reduction-sensitive or reduction-insensitive) at 37°C. At 0, 3, 12 h, 40 μL samples were taken and added to 160 μL of ACN for analysis. The content change of the PPa-S-S-TG was monitored by HPLC. The PPa-S-S-TG and the PPa solution (10 mg/kg equivalent to PPa) were administrated to female BALB/c mice bearing the 4T1 cell line. At scheduled times the mice were sacrificed and the GITs, MLNs and tumors were taken out for fluorescence imaging by the Caliper IVIS ® Spectrum in vivo Imaging System. The intestinal tissue of PPa-S-S-TG group was then washed, fixed with 4% paraformaldehyde for 2 h, and dehydrated in 30% sucrose overnight. Then the tissue was frozen in optimal cutting temperature-compound at -20 °C, sectioned into 10 μm intervals and stained with Hoechst 33258. Finally, the tissue-sections were observed using a CLSM.

***In vivo antitumor effect***

The antitumor effect of the TG prodrugs was investigated in female BALB/c mice bearing the 4T1 cell line. In brief, a 0.2-mL cell suspension containing $5\times10^6$ 4T1 cells

were injected subcutaneously into the flank of the mice. When the tumor reached 150 $mm^3$, the mice were administered orally with the DTX solution at a dose of 20 mg/kg or the prodrug emulsions at a dose of 20 mg/kg equivalent to DTX or given intravenously with the DTX solution at a dose of 10 mg/kg (n=5 each group). Saline was used as a control. The mice were treated with the above preparations every alternate day for 10 days. Body weight and tumor volume were recorded every day. At day 10, the mice were sacrificed and major organs and tumors were collected and fixed in 4% paraformaldehyde solution for hematoxylin and eosin (H&E) staining.

***Gastrointestinal toxicity evaluation***

Nine female BALB/c mice were divided into four groups (n=3 each group) and were orally administered the DTX solution (20 mg/kg), DTX-S-S-TG prodrug emulsion (20 mg/kg equivalent to DTX) or intravenous injection with DTX solution (10 mg/kg) and saline (control) every day. Five days later, the mice were sacrificed and their blood was obtained for hepatic and renal function analysis. The stomach, duodenum, jejunum and ileum were taken and fixed in 4% paraformaldehyde solution for hematoxylin and eosin (H&E) staining. The histological sections were imaged using an optical microscope.

***Statistical analysis***

Student's t-test was used to determine significant differences between the two groups. When $p<0.05$, there was a significant difference, and very significant differences between two groups were defined as $p<0.01$.

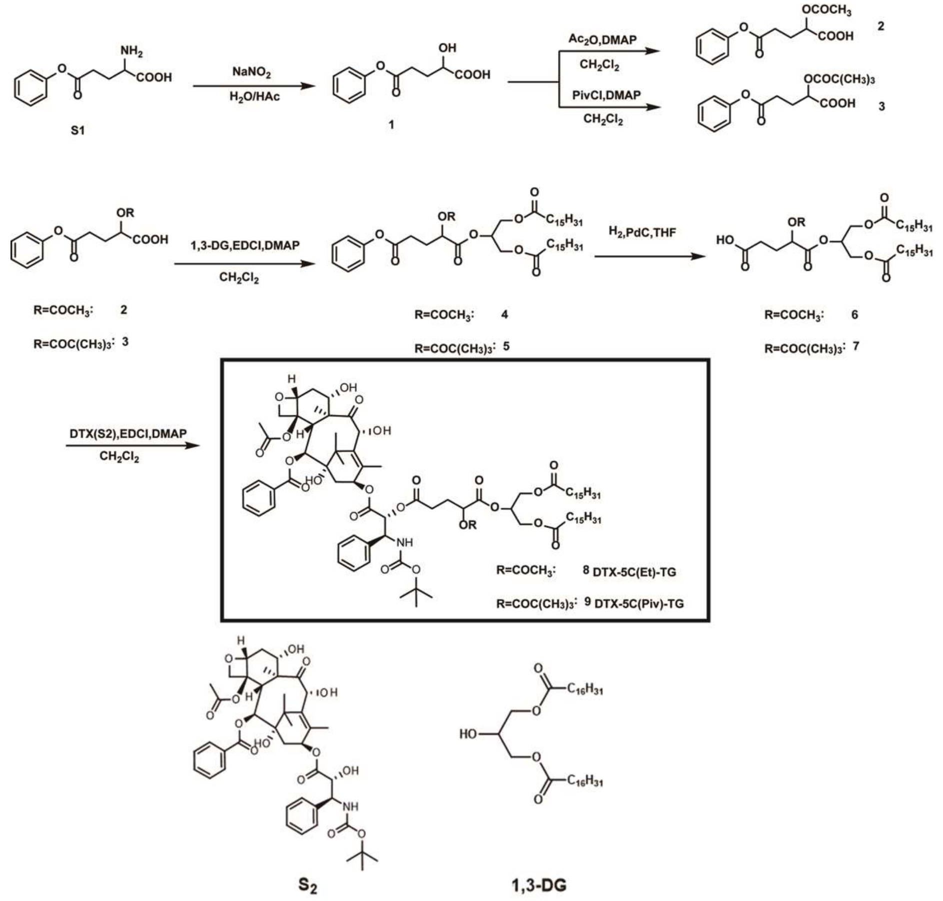
NaNO2
H2O/HAc
Ac2O,DMAP
CH2Cl2
PivCl,DMAP
CH2Cl2
S1
1
2
3
1,3-DG,EDCI,DMAP
CH2Cl2
H2,PdC,THF
R=COCH3: 2
R=COC(CH3)3: 3
R=COCH3: 4
R=COC(CH3)3: 5
R=COCH3: 6
R=COC(CH3)3: 7
DTX(S2),EDCI,DMAP
CH2Cl2
R=COCH3: 8 DTX-5C(Et)-TG
R=COC(CH3)3: 9 DTX-5C(Piv)-TG
S2
1,3-DG

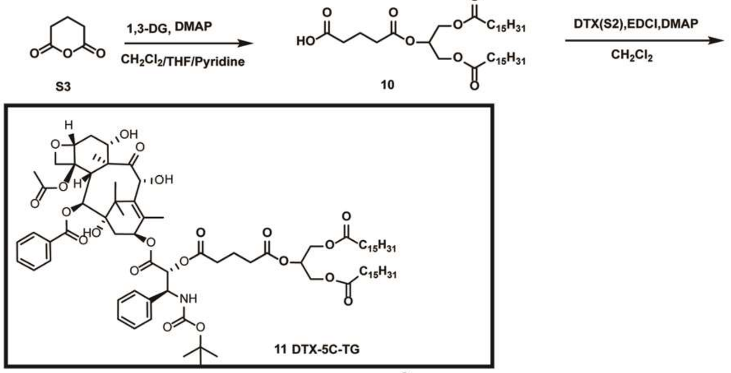
1,3-DG, DMAP
CH2Cl2/THF/Pyridine
S3
10
DTX(S2),EDCI,DMAP
CH2Cl2
11 DTX-5C-TG

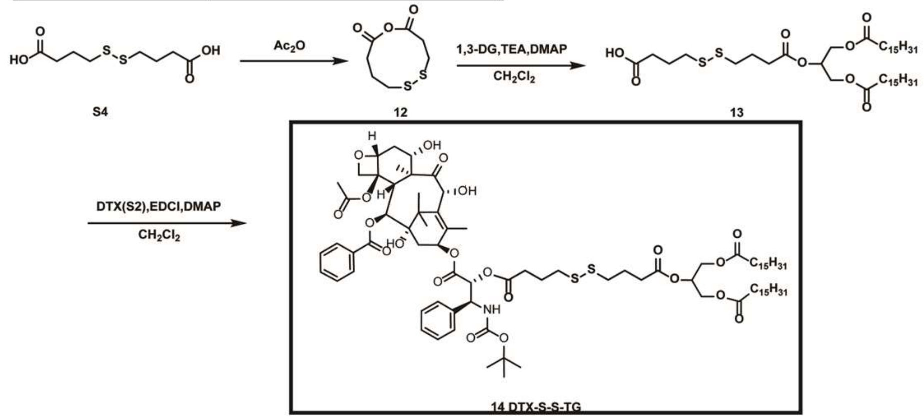
Ac2O
S4
12
1,3-DG,TEA,DMAP
CH2Cl2
13
DTX(S2),EDCI,DMAP
CH2Cl2
14 DTX-S-S-TG

**Scheme S1.** The synthetic route of the designed triglyceride-mimetic prodrugs.

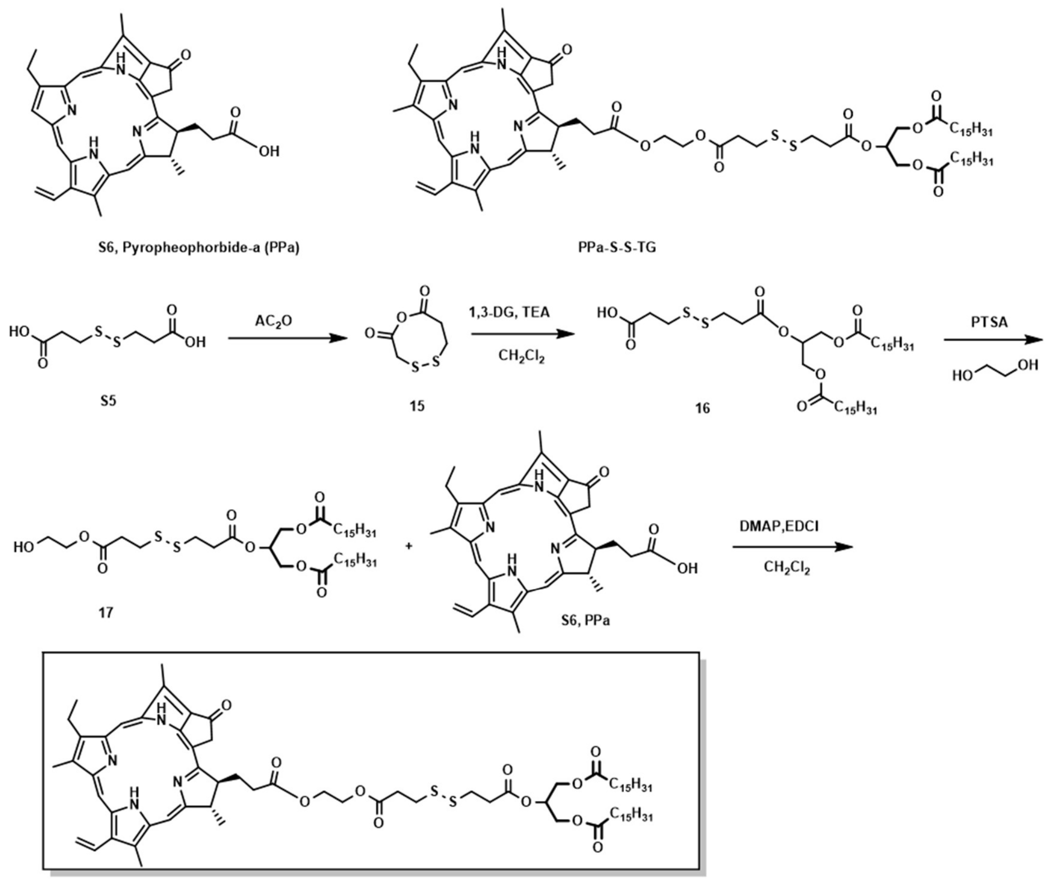


**Scheme S2.** The synthetic route of the designed triglyceride-mimetic dye.

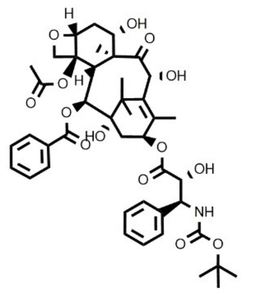

DTX cLogP=4.22

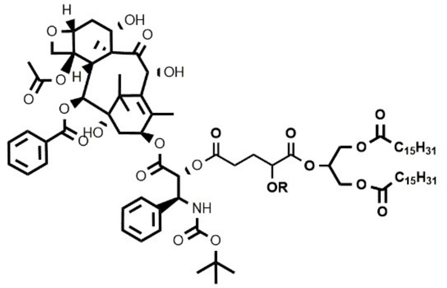

R=$COCH_3$: DTX-5C(Et)-TG cLogP=15.86

R=$COC(CH_3)_3$: DTX-5C(Piv)-TG cLogP=17.66

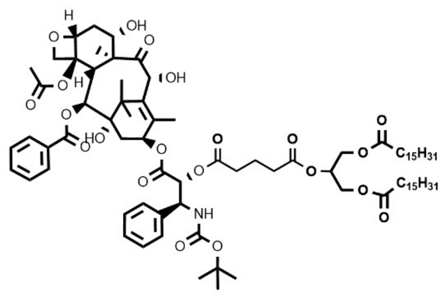

DTX-5C-TG cLogP=16.29

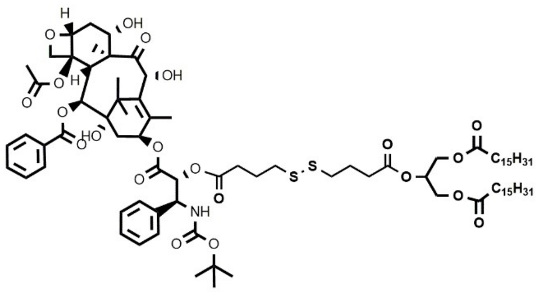

DTX-S-S-TG cLogP=17.55

**Figure S1.** Chemical structures of DTX and its triglyceride-mimetic prodrugs and their cLogpP calculated by MarvinSketch software.

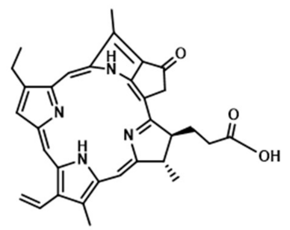

Pyropheophorbide-a (PPa)

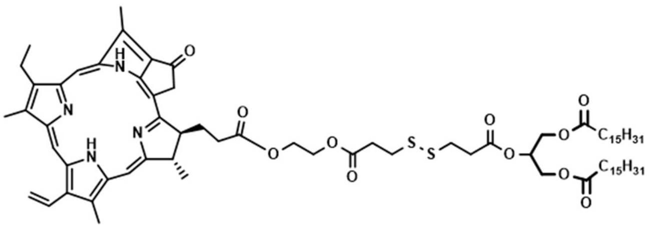

PPa-S-S-TG

**Figure S2.** Chemical structures of PPa and its triglyceride-mimetic dye.

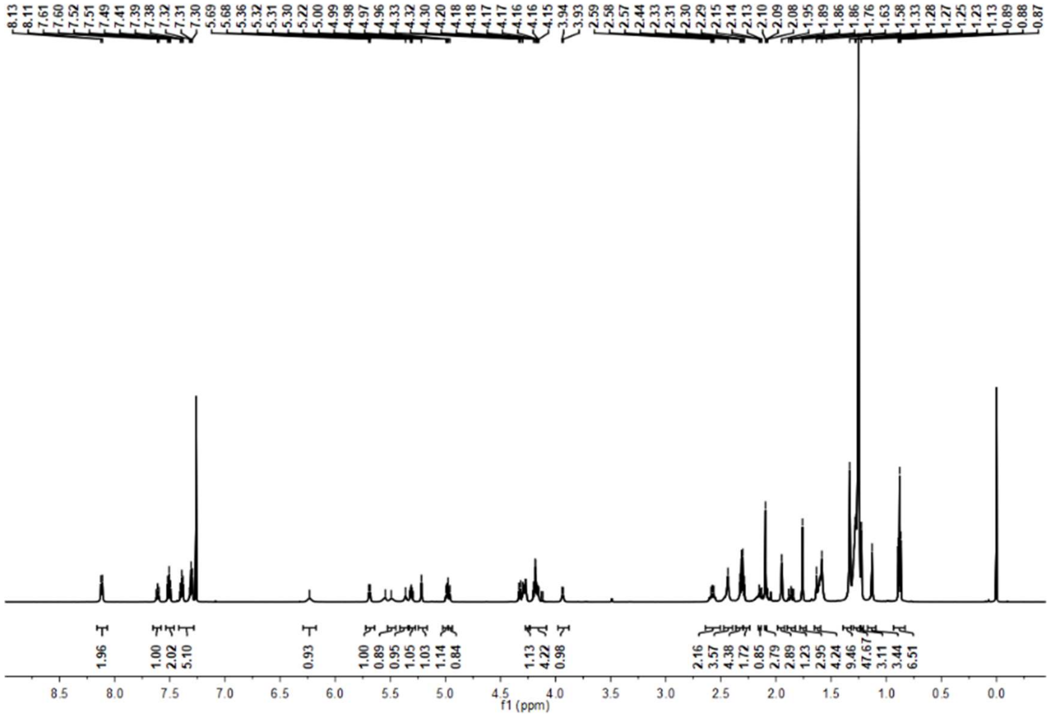


**Figure S2.** $^{1}$H-NMR spectra of DTX-5C(Et)-TG.

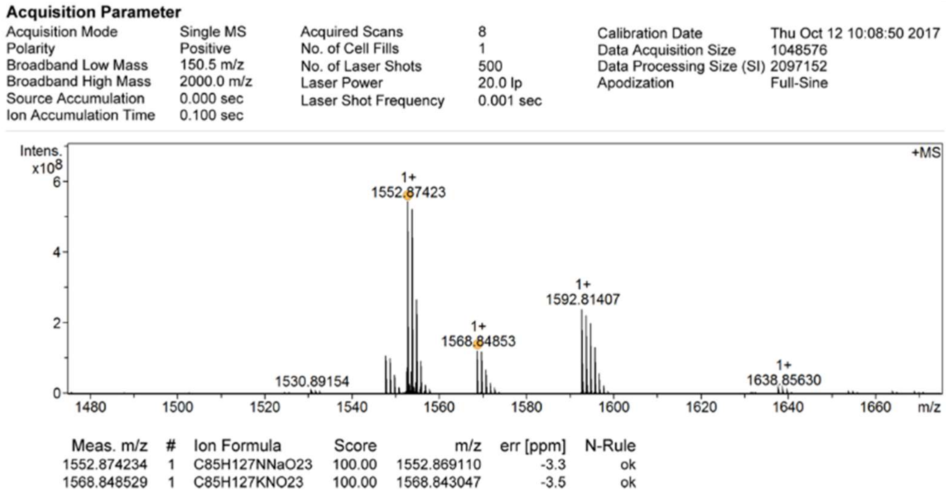


**Figure S3.** Mass spectra of DTX-5C(Et)-TG.

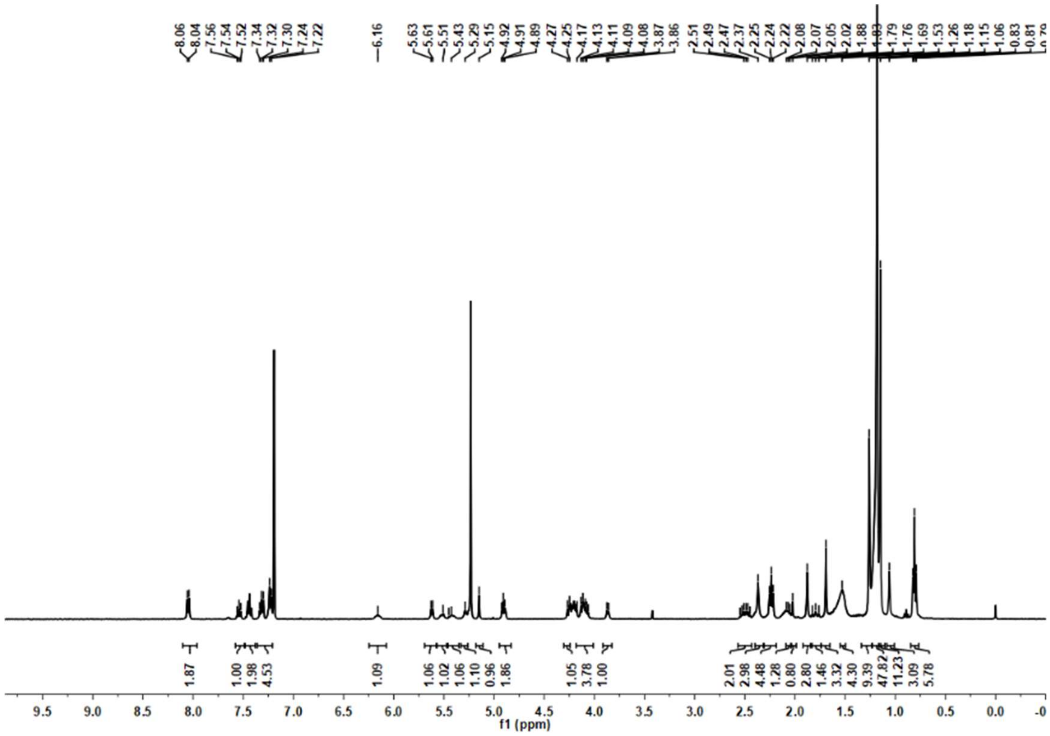


**Figure S4.** $^{1}$H-NMR spectra of DTX-5C(Piv)-TG.

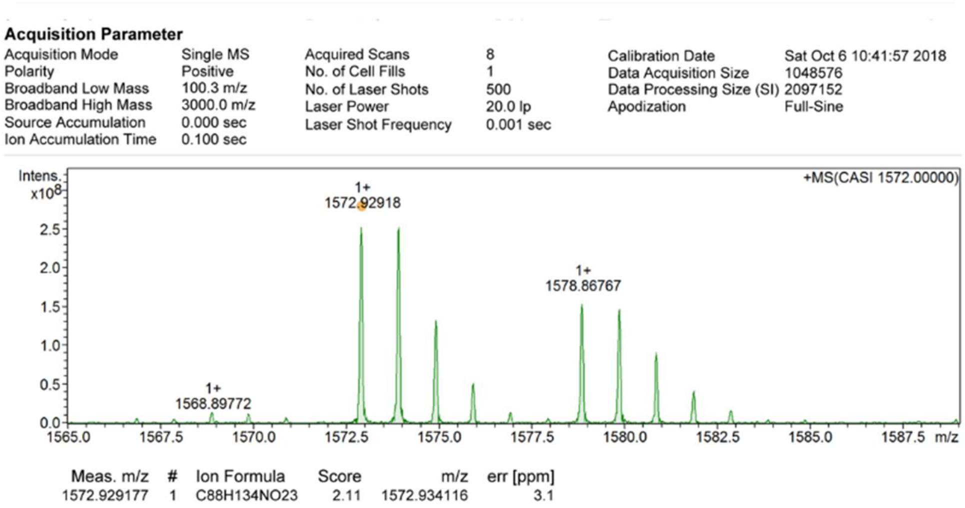


**Figure S5.** Mass spectra of DTX-5C(Piv)-TG.

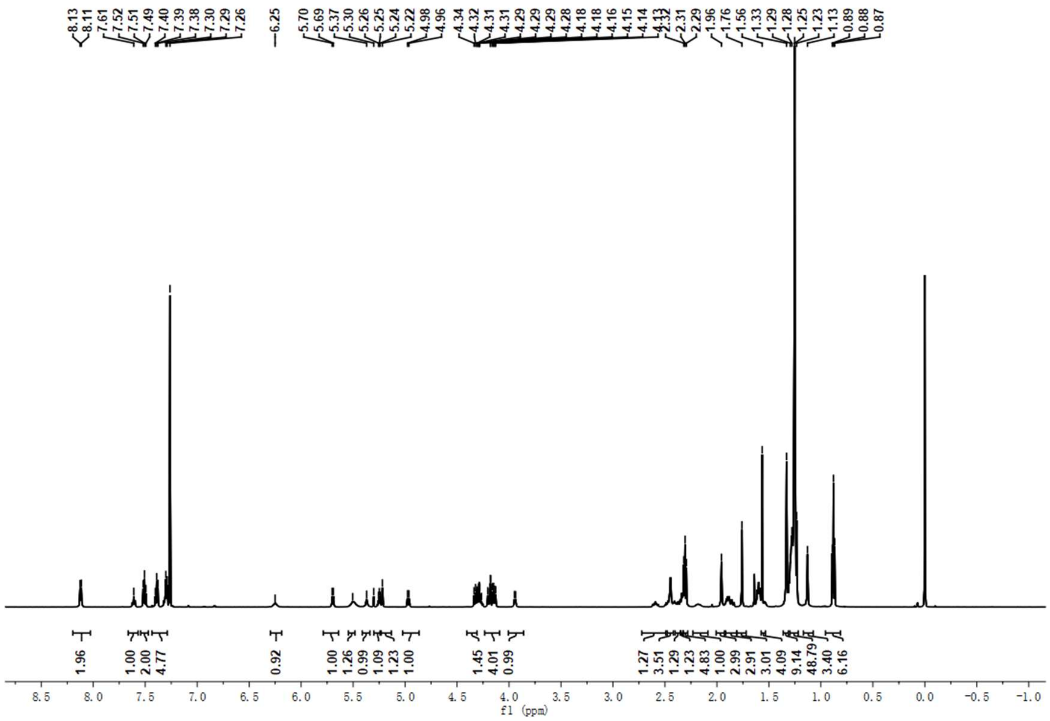


**Figure S6.** $^{1}$H-NMR spectra of DTX-5C-TG.

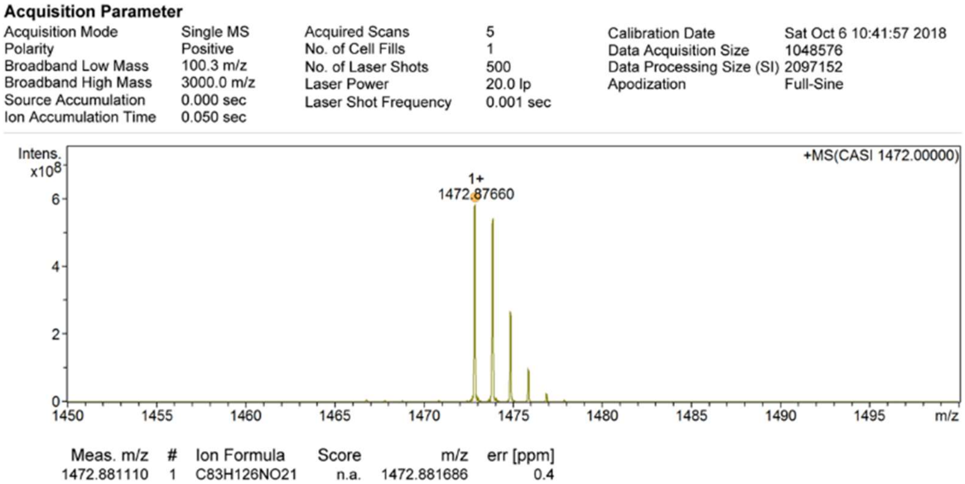


**Figure S7.** Mass spectra of DTX-5C-TG.

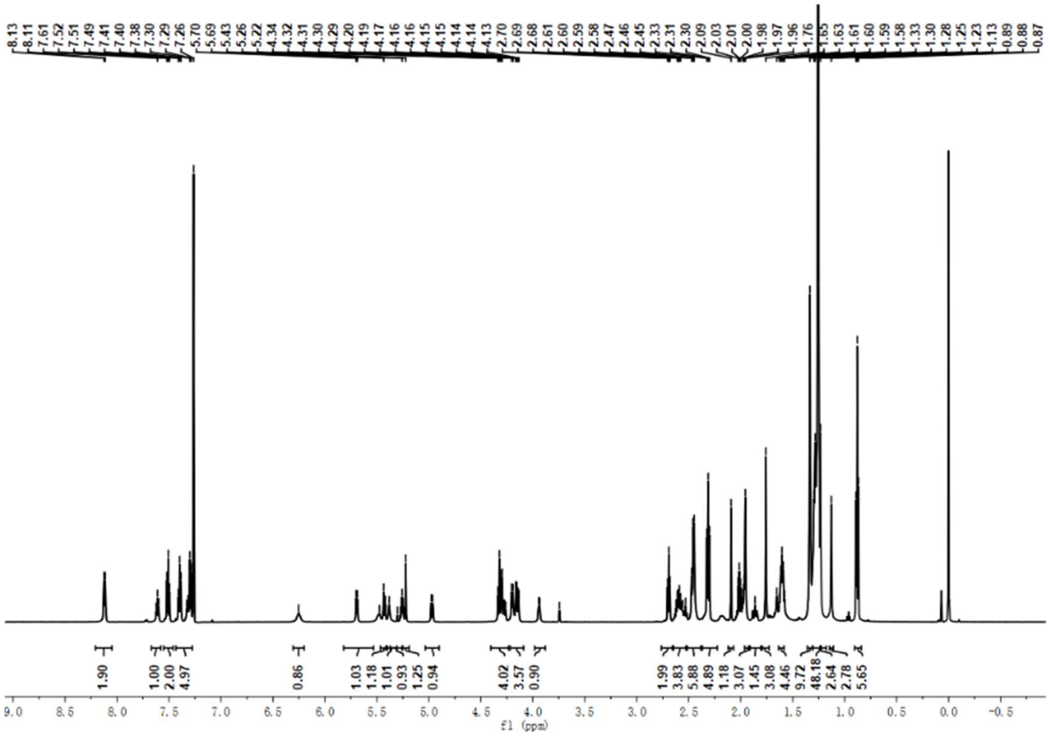


**Figure S8.** $^{1}$H-NMR spectra of DTX-S-S-TG.

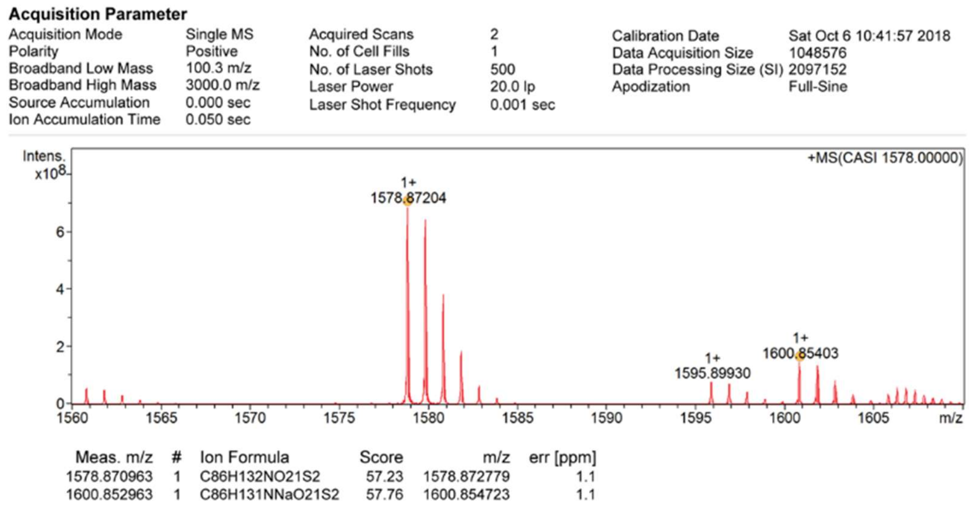


**Figure S9.** Mass spectra of DTX-S-S-TG.

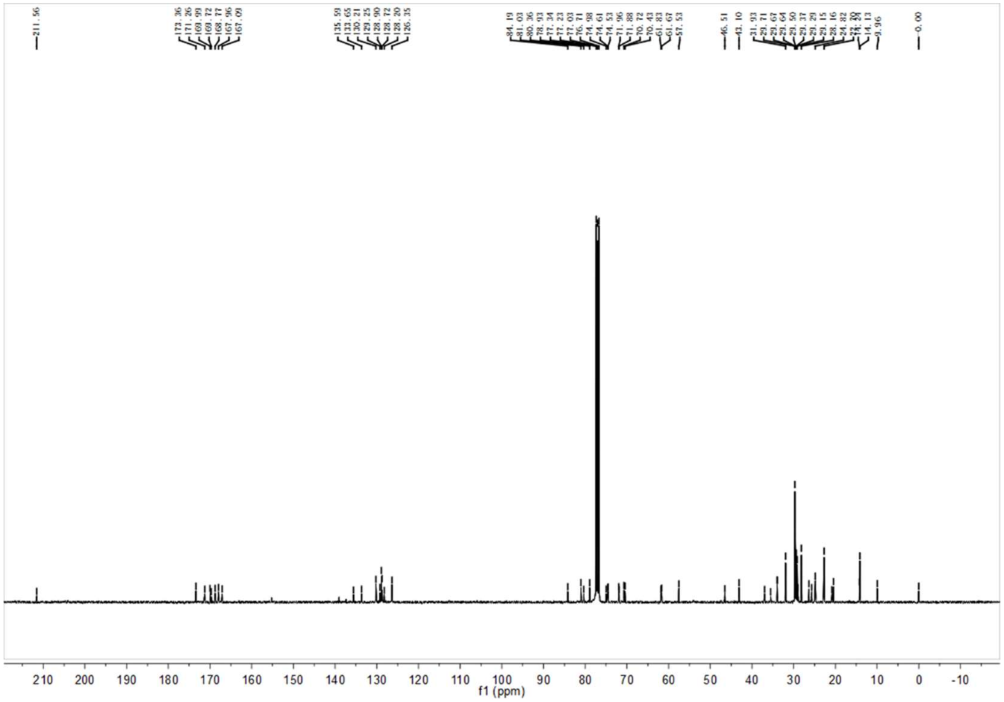


**Figure S10.** $^{13}$C-NMR spectra of DTX-5C(Et)-TG.

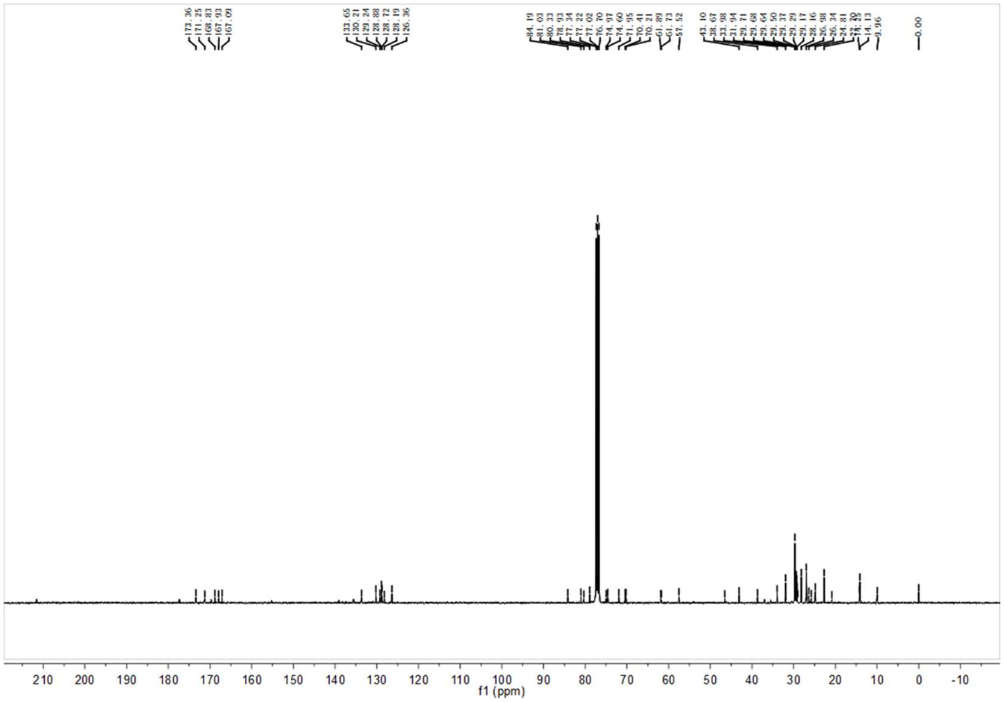


**Figure S11.** $^{13}$C-NMR spectra of DTX-5C(Piv)-TG.

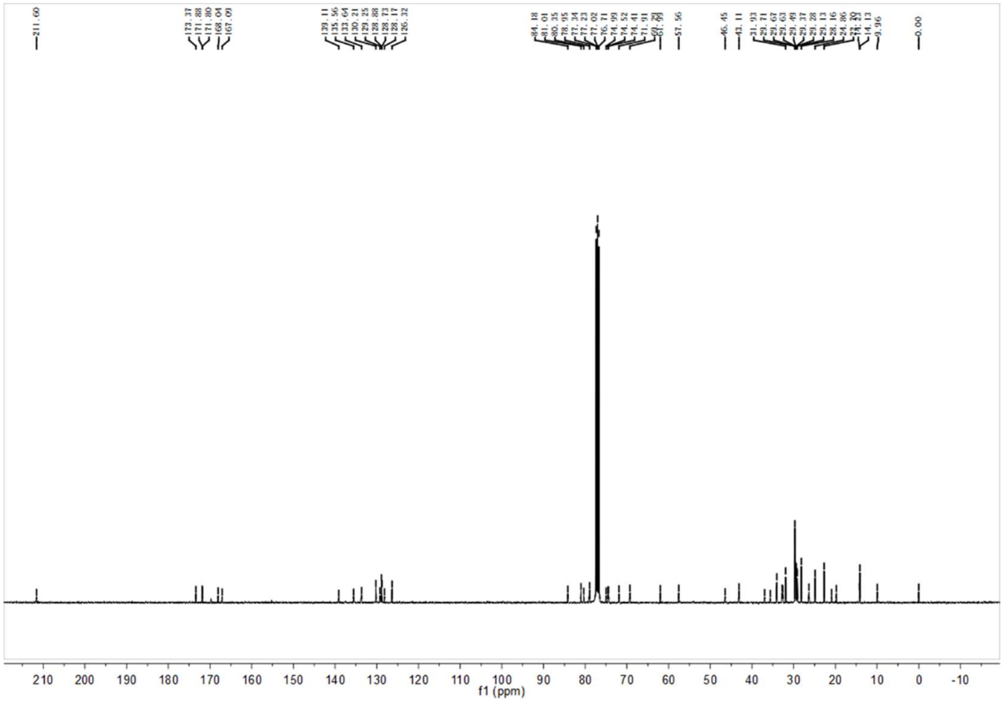

**Figure S12.** $^{13}$C-NMR spectra of DTX-5C-TG.

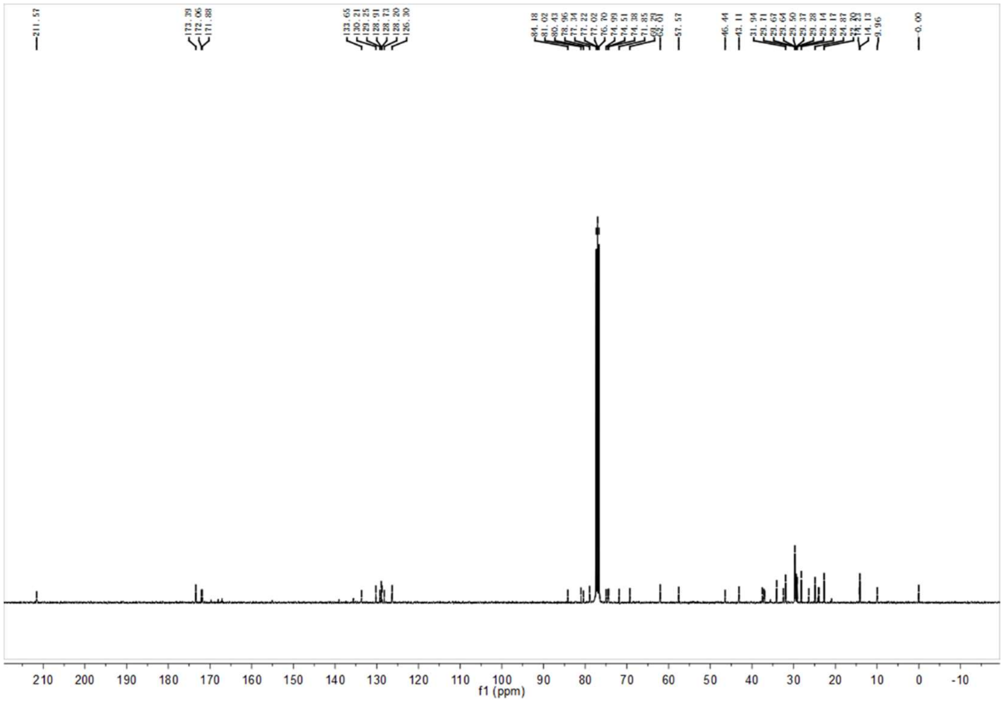

**Figure S13.** $^{13}$C-NMR spectra of DTX-S-S-TG.

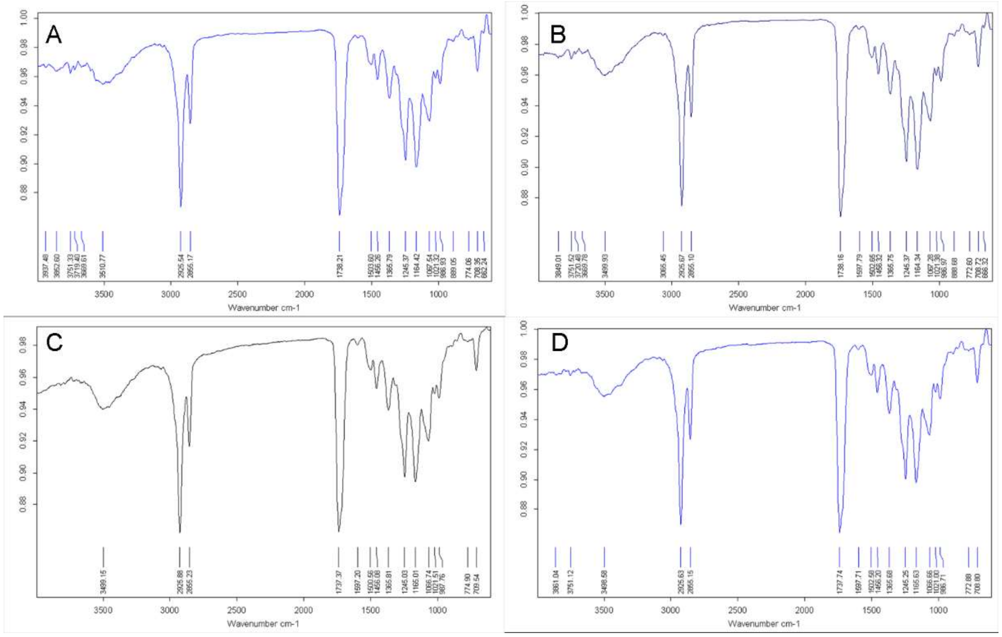


**Figure S14.** IR spectra of (A) DTX-5C(Et)-TG. (B) DTX-5C(Piv)-TG. (C) DTX-5C-TG. (D) DTX-S-S-TG.

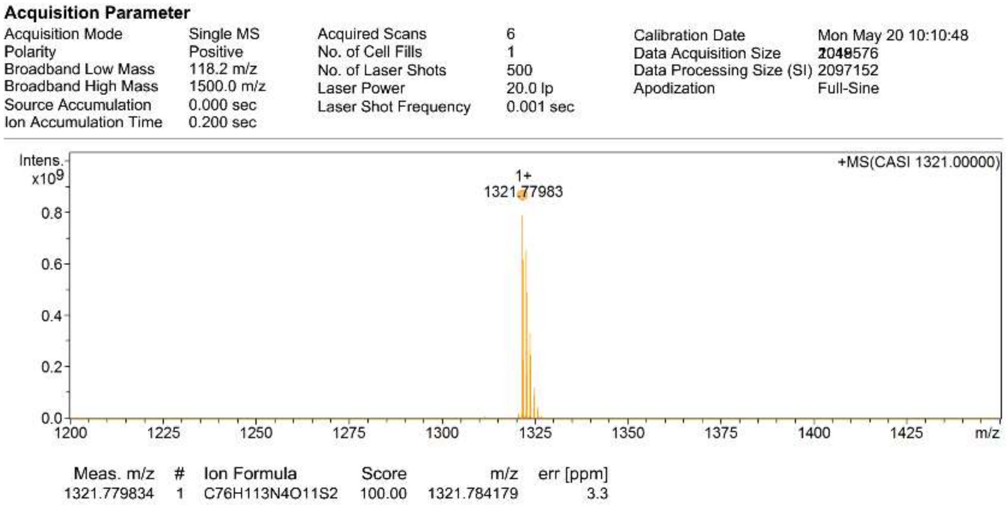


**Figure S15.** Mass spectra of PPa-S-S-TG.

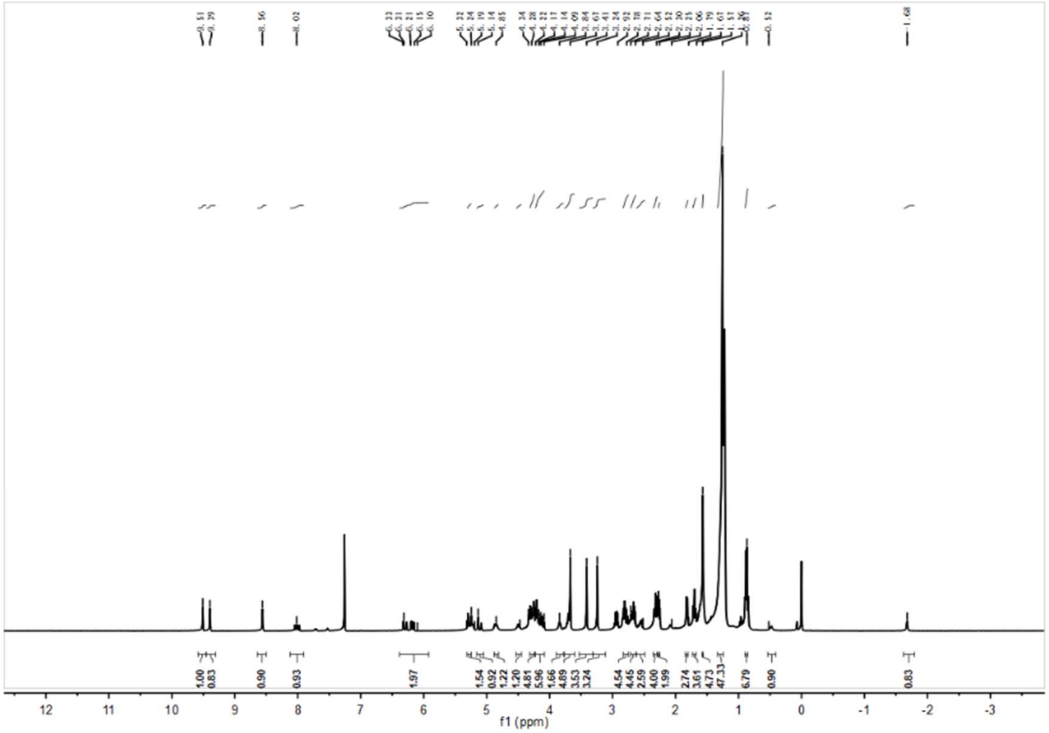

**Figure S16.** $^{1}$H-NMR spectra of PPa-S-S-TG.

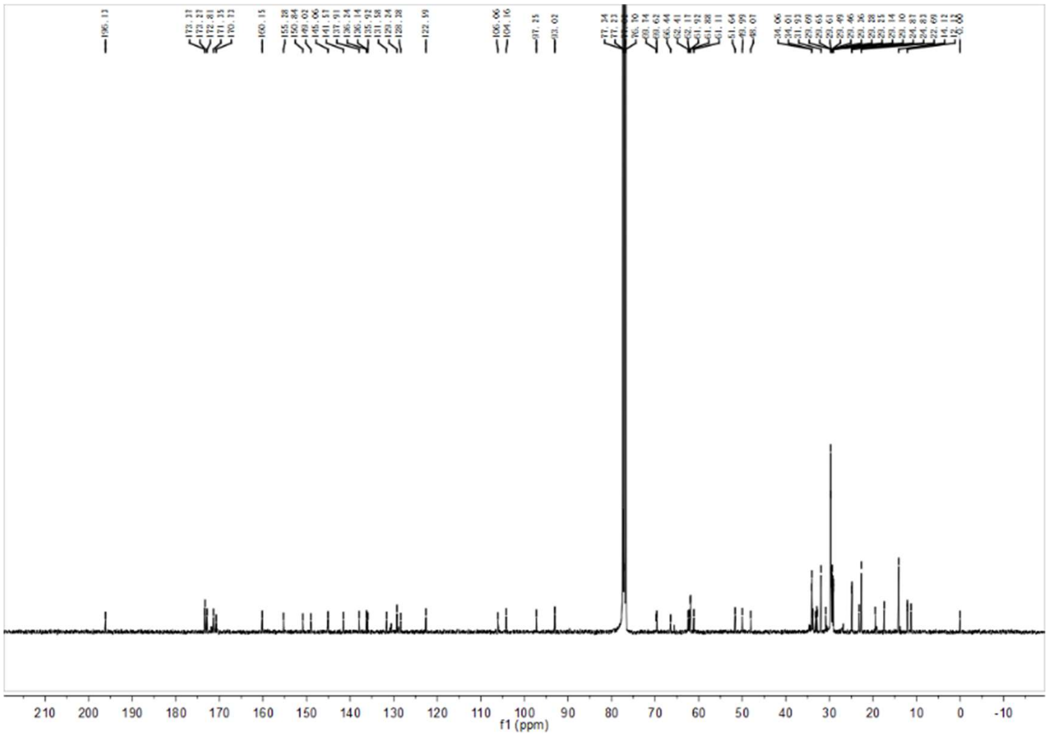

**Figure S17.** $^{13}$C-NMR spectra of PPa-S-S-TG.

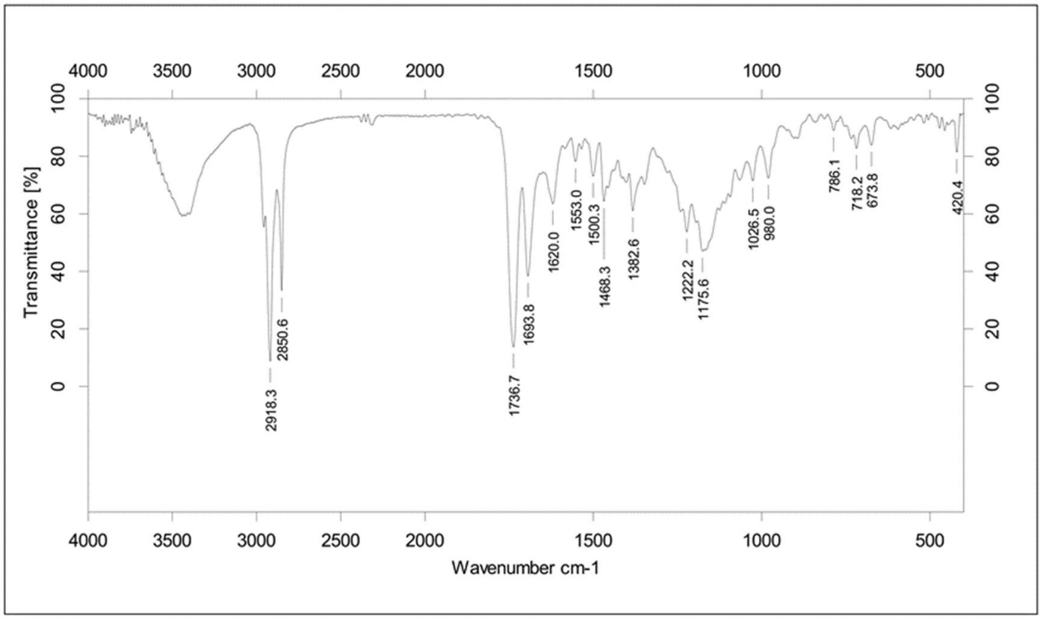


**Figure S18.** The infrared spectroscopy of PPa-S-S-TG.

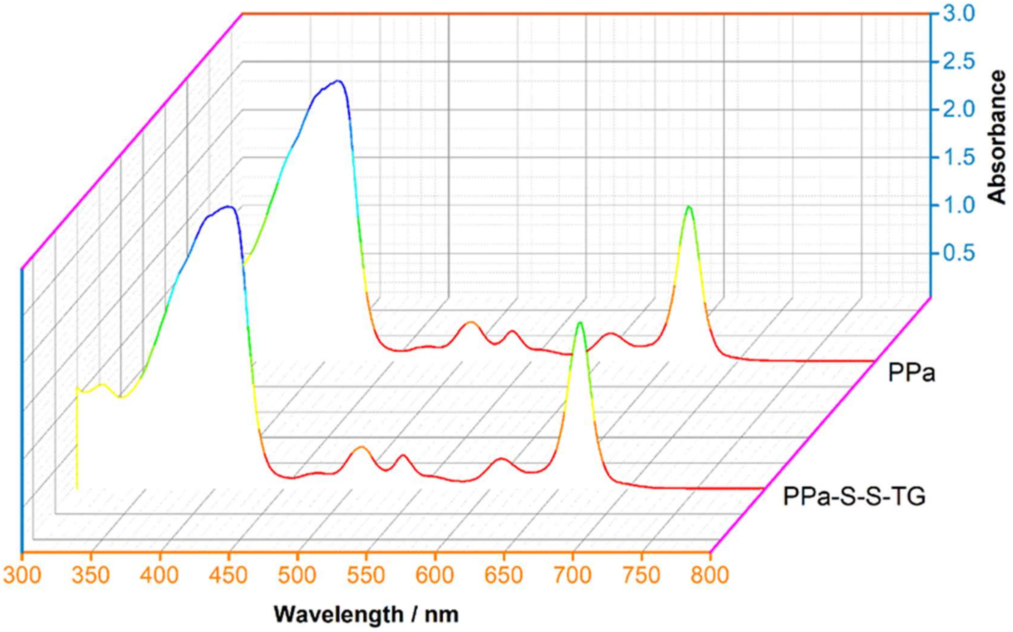


**Figure S19.** Ultraviolet spectra of PPa, PPa-S-S-TG dissolved in acetonitrile.

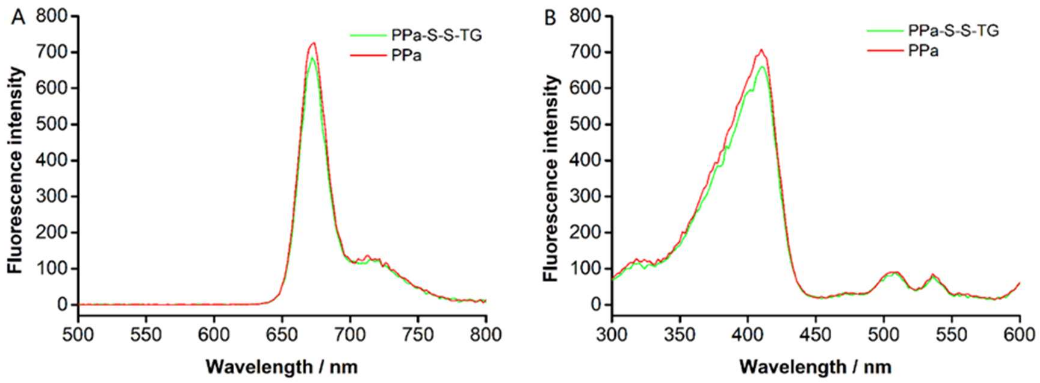


**Figure S20.** Fluorescence spectra of PPa and PPa-S-S-TG dissolved in acetonitrile. (A) Excitation spectra at a fixed emission of 415 nm; (B) Emission spectra at a fixed excitation of 675 nm.

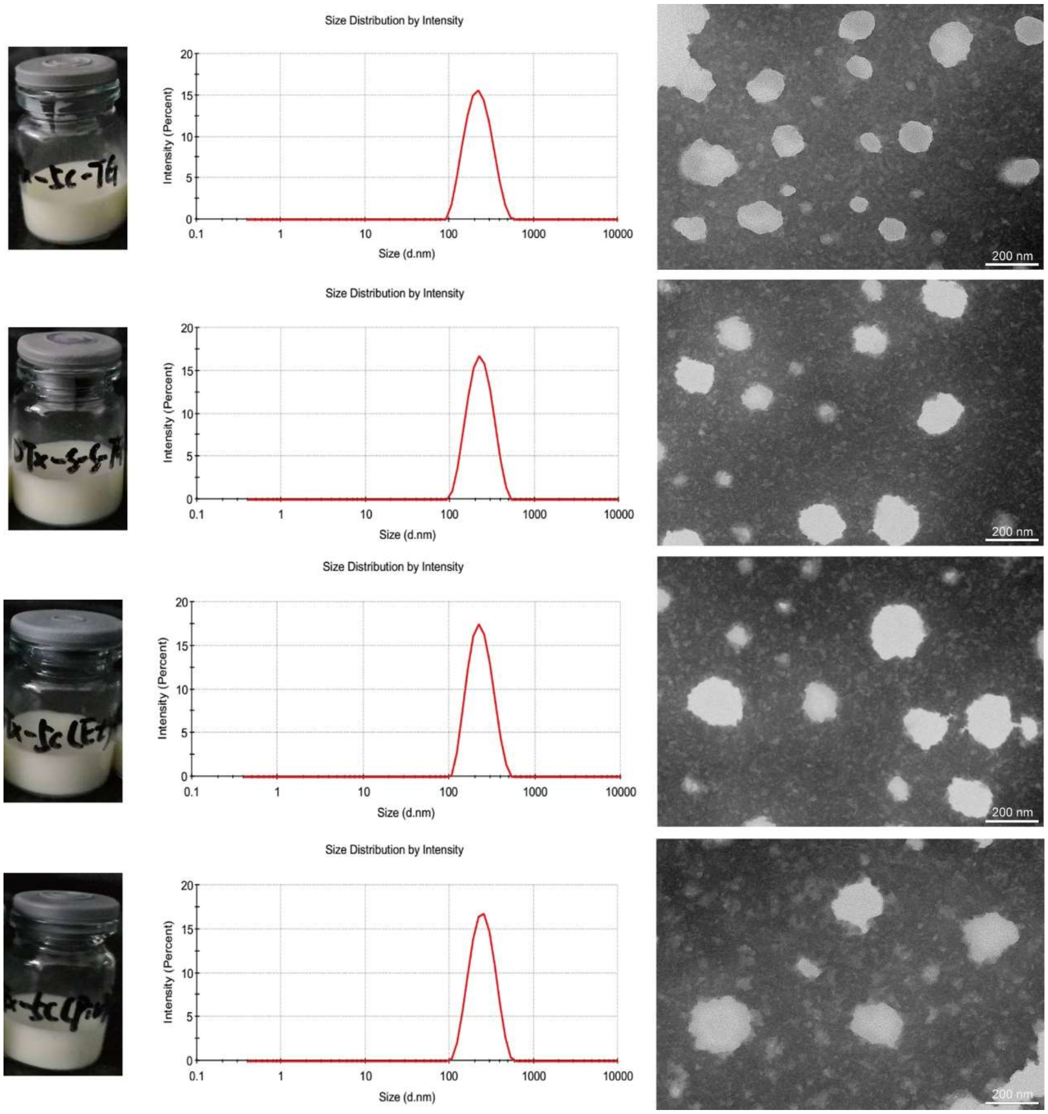


**Figure S21.** Appearance, size distribution and TEM images of chylomicron-like emulsions.

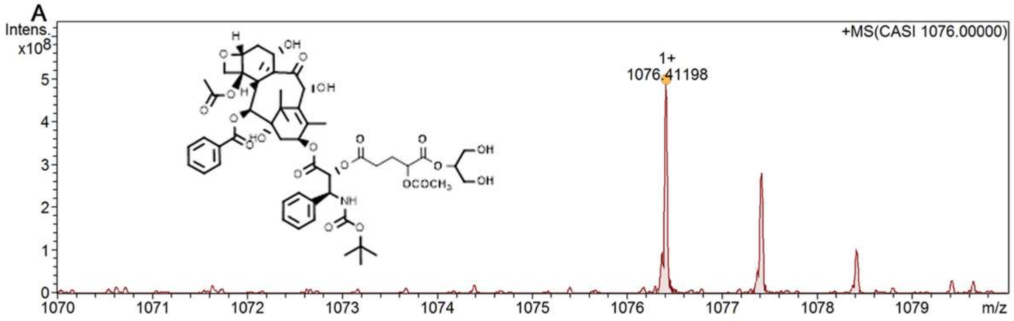


| Meas. m/z | # | Ion Formula | Score | m/z | err [ppm] |
|---|---|---|---|---|---|
| 1076.411982 | 1 | C53H67NNaO21 | 100.00 | 1076.409779 | -2.0 |

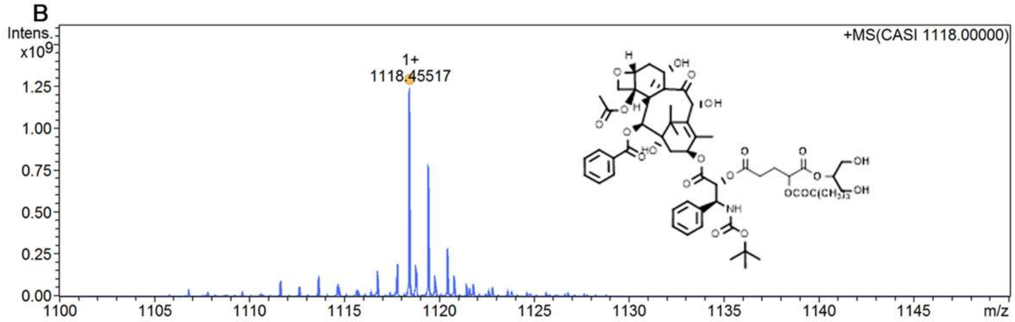


| Meas. m/z | # | Ion Formula | Score | m/z | err [ppm] |
|---|---|---|---|---|---|
| 1118.455169 | 1 | C56H73NNaO21 | 100.00 | 1118.456729 | 1.4 |

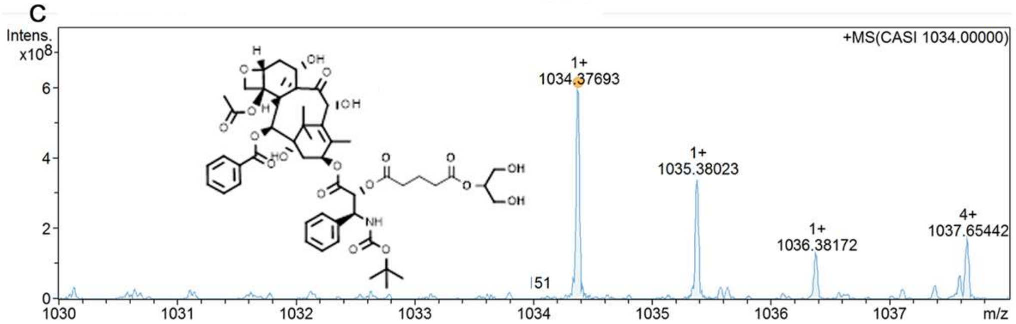


| Meas. m/z | # | Ion Formula | Score | m/z | err [ppm] |
|---|---|---|---|---|---|
| 1034.376930 | 1 | C51H65KNO19 | 100.00 | 1034.378237 | 1.3 |

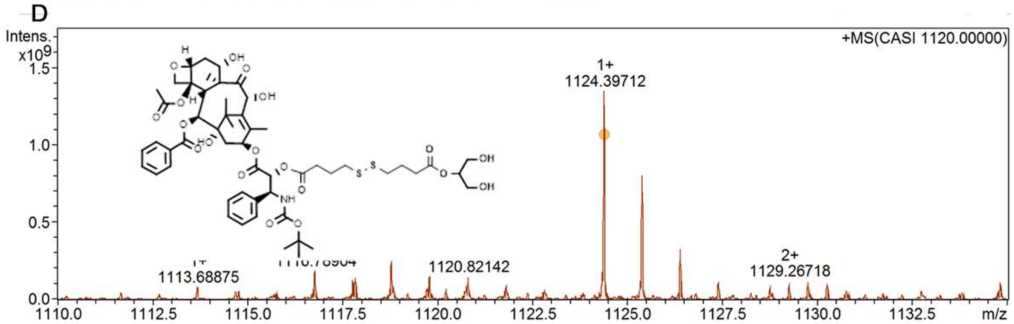


| Meas. m/z | # | Ion Formula | Score | m/z | err [ppm] | Mean err [ppm] |
|---|---|---|---|---|---|---|
| 1124.397125 | 1 | C54H71NNaO19S2 | 66.17 | 1124.395392 | -1.5 | -0.5 |

**Figure S22.** Mass spectra of (A) DTX-5C(Et)-MG (B) DTX-5C(Piv)-MG (C) DTX-5C-MG (D) DTX-S-S-MG.

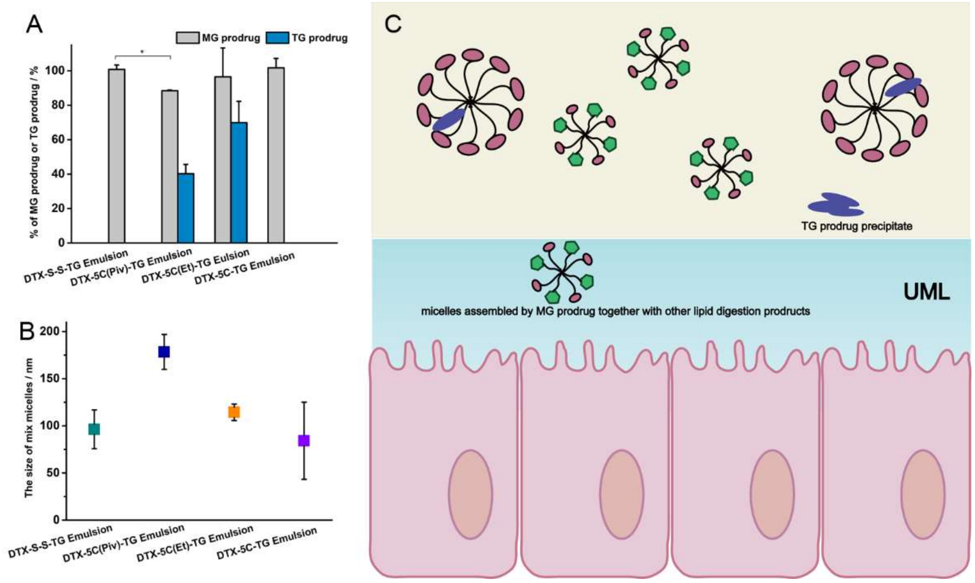


**Figure S23.** (A) The fraction of TG-like prodrugs or MG-like prodrugs partitioning in the mixed micellar phase after 3h digestion of four prodrugs. (B) The particle size of the mixed micelles formed after 3h digestion of four prodrugs. (C) Schematic representation illustrating the possible fate of TG-like prodrugs after digestion in intestinal lumen. (Data are represented as mean ± SD, n=3, *P <0.05, the inset P values indicate the significance between groups).

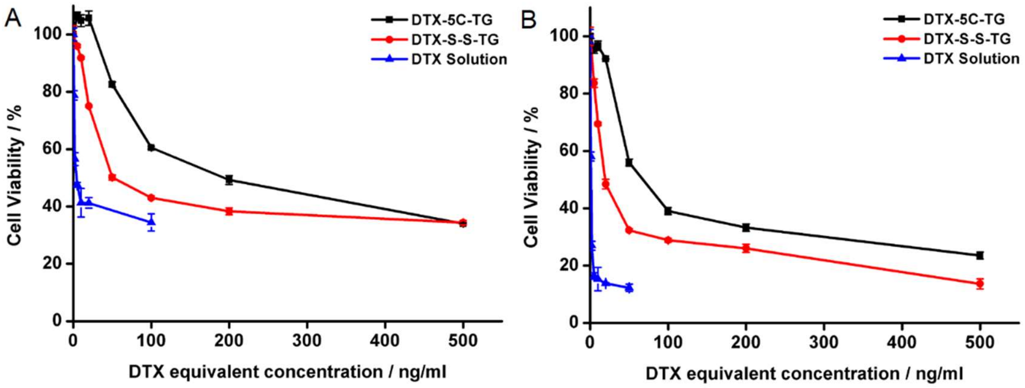


**Figure S24.** Cell viability of KB cells after treated with various concentration of DTX solution, DTX-S-S-TG prodrug and DTX-5C-TG prodrug for (A) 48 h (B) 72 h.

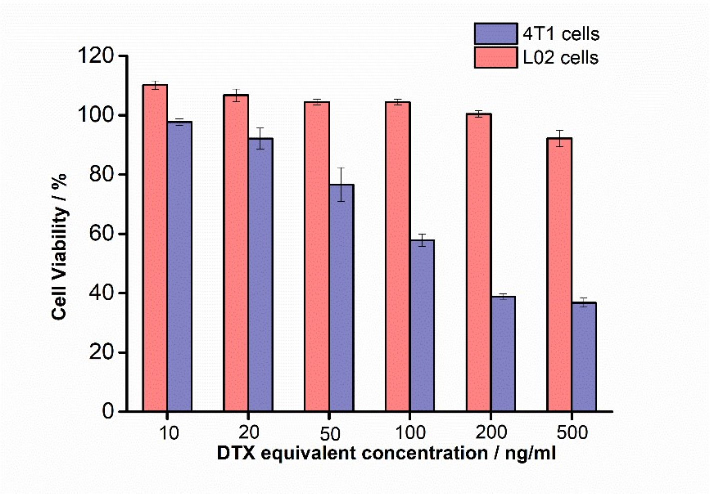


**Figure S25.** Cell viability of 4T1 and L02 cells after treated with various concentration of DTX-S-S-TG prodrug for 48 h.

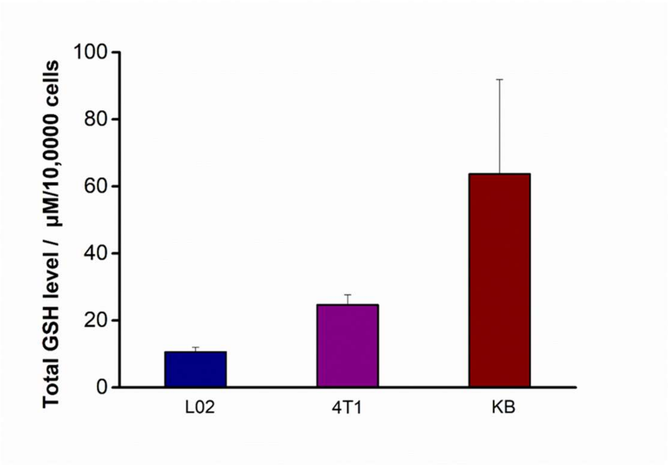


**Figure S26.** Total cellular GSH level of L02, 4T1 and KB cells.

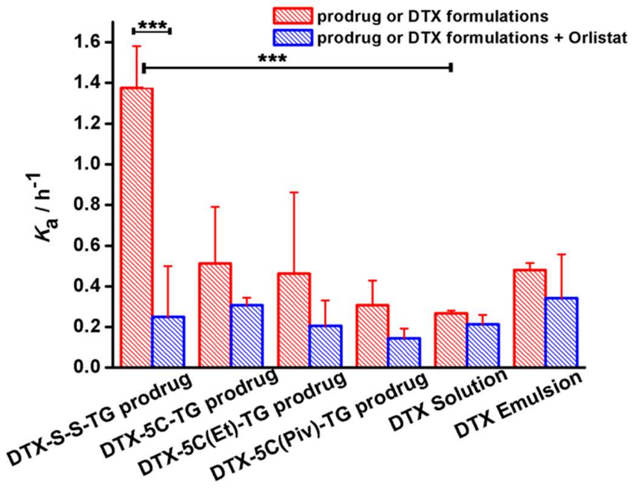


**Figure S27.** *K*a values of TG prodrugs and DTX formulations and effect of orlistat on membrane permeability. (data are represented as mean ± SD, n=3, ***P <0.001, The inset P values indicate the significance between groups).

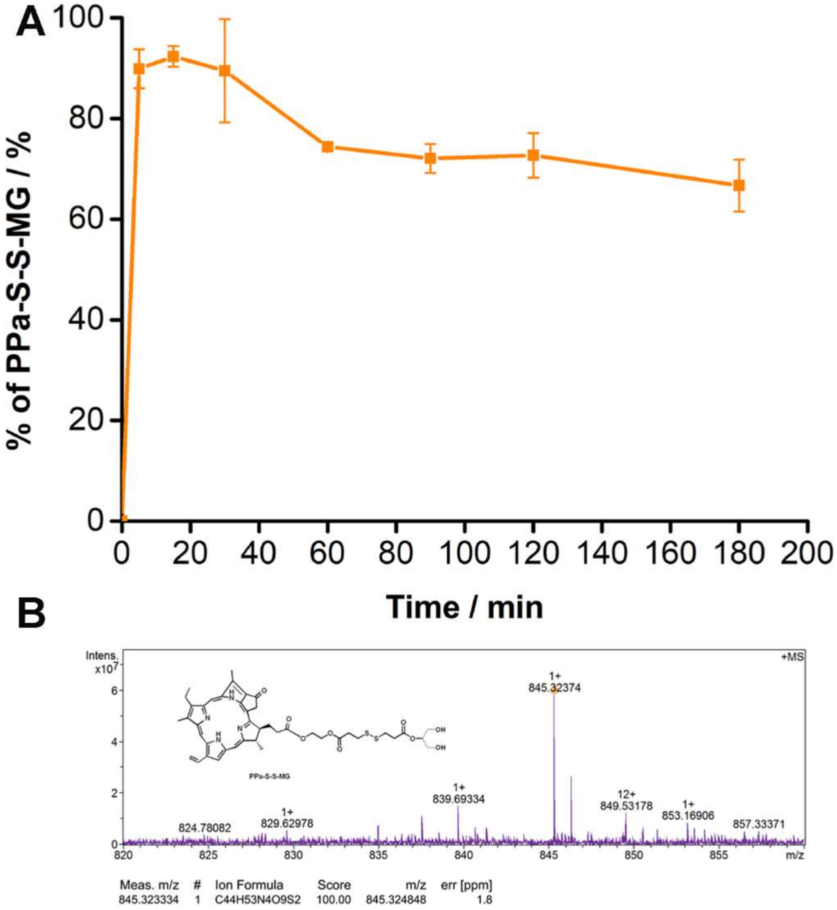


**Figure S28.** Simulated intestinal digestion of the PPa-S-S-TG. (A) Generation of MG prodrugs after incubation with bile-pancreatic juice. (B) Mass spectra of the PPa-S-S-MG.

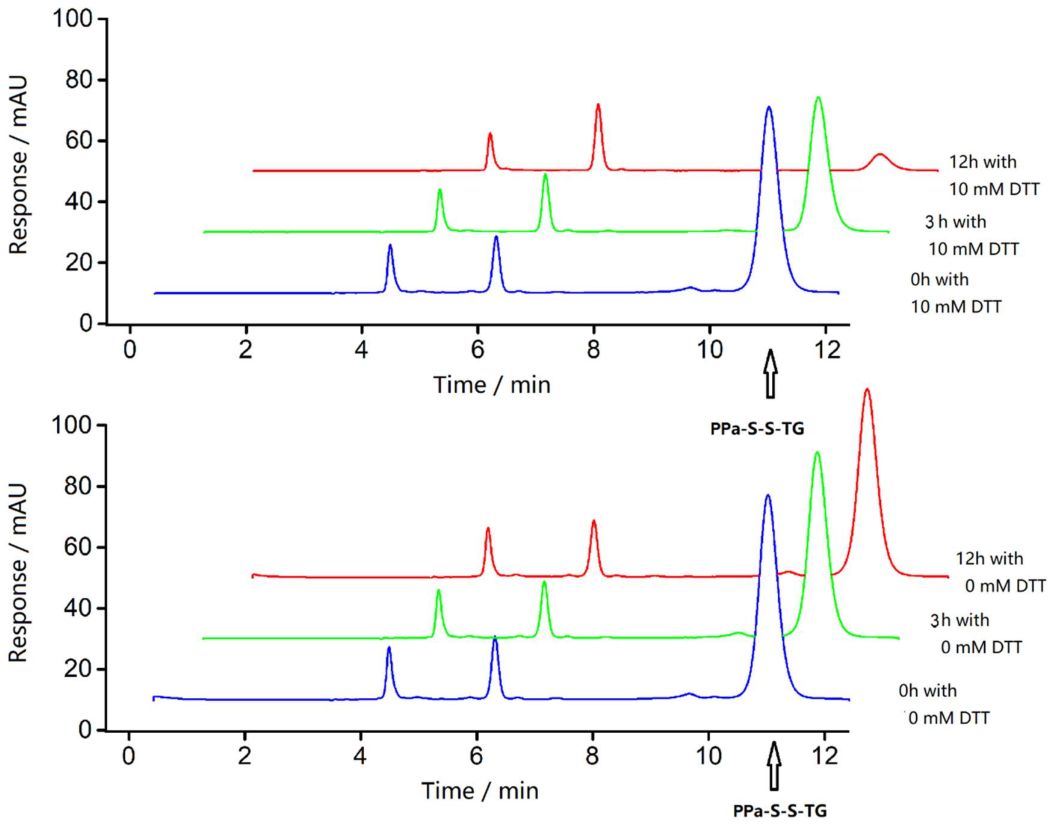


**Figure S29.** The content change of the PPa-S-S-TG incubation with or without DTT.

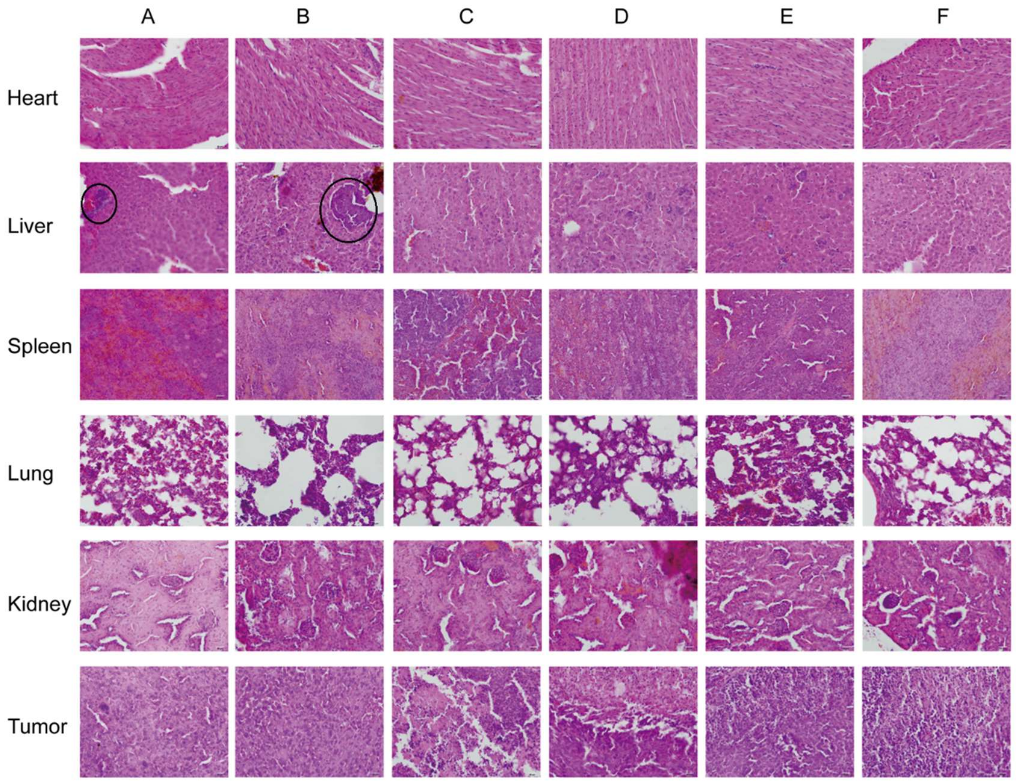


**Figure S30.** H&E staining of the major organs and tumors after treatments. (A) NS (B) DTX Solution (i.g.) (C) DTX Solution (i.v.) (D) DTX-S-S-TG (E) DTX-5C-TG (F) DTX-5C(Et)-TG.

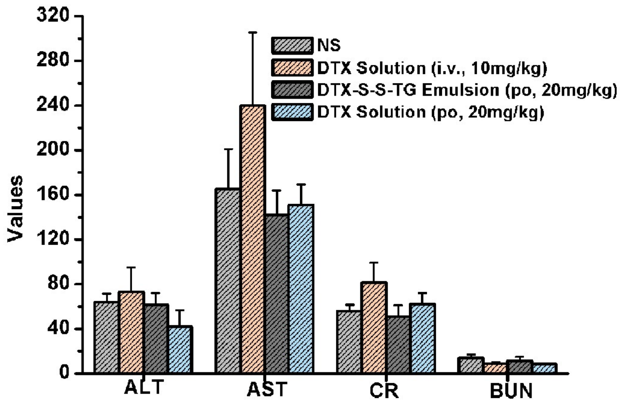


**Figure S31.** Hepatic and renal function indicators of mice after treatment (data are presented as mean ± SD, n = 3).

**Table S1.** Cytotoxicity ($IC_{50}$ values) of DTX and prodrugs to three tumor cell lines.

| Formulations | 4T1 (ng/mL) | | KB (ng/mL) | |
|---|---|---|---|---|
| | 48 h | 72 h | 48 h | 72 h |
| DTX Solution | 16.6 | 5.9 | 4.9 | 1.3 |
| DTX-S-S-TG | 177.4 | 31.6 | 99.9 | 26.8 |
| DTX-5C-TG | 357.1 | 156.9 | 226.5 | 88.2 |

**References**


[1] P. H. Bentley, W. Mccrae, *Journal of Organic Chemistry* **1970**, 35, 2082.

[2] W. Song, Y. Yang, M. Yu, Q. Zhu, H. Zhong, Y. Gan, *Asian Journal of Pharmaceutical Sciences* **2018**, 13.

[3] W. Zhang, C. Liang, H. Liu, Z. Li, R. Chen, M. Zhou, D. Li, Q. Ye, C. Luo, J. Sun, *Asian Journal of Pharmaceutical Sciences* **2017**, 12, 586.

[4] V. H. Levan, G. M. Green, Effect of Diversion of Bile-Pancreatic Juice to the Ileum on Pancreatic Secretion and Adaptation in the Rat, *Experimental Biology and Medicine* **1986**, 181, 139.

[5] M. Grassi, G. Cadelli, *International Journal of Pharmaceutics* **2001**, 229, 95.
